# Oral bacterial community dynamics during induction of gingival inflammation

**DOI:** 10.3389/fcimb.2025.1597690

**Published:** 2025-06-16

**Authors:** Bart J. F. Keijser, Tim J. van den Broek, Michelle van der Wurff, Remon Dulos, Ferry Jagers, Jolanda Kool, Martijn Rosema, Elena A. Nicu, Wim Crielaard, Bruno G. Loos, Bernd W. Brandt, Egija Zaura

**Affiliations:** ^1^ Research Group Microbiology and Systems Biology, TNO, Leiden, Netherlands; ^2^ Department of Preventive Dentistry, Academic Centre for Dentistry Amsterdam, University of Amsterdam and Vrije Universiteit Amsterdam, Amsterdam, Netherlands; ^3^ Department of Periodontology, Academic Centre for Dentistry Amsterdam, University of Amsterdam and Vrije Universiteit Amsterdam, Amsterdam, Netherlands

**Keywords:** oral microbiota, microbial dynamics, gingivitis, plaque, dysbiosis, 16S rRNA gene sequencing, inflammation

## Abstract

**Introduction:**

The human oral cavity is a complex and dynamic microbial ecosystem integral to oral and overall health. While the specific roles of microbial communities in health and disease are not fully understood, dysbiosis of the oral microbiota is, along with inadequate immune fitness, recognized as a key factor driving the onset of inflammatory conditions such as gingivitis. Gingivitis, an early and reversible stage of periodontal disease, involves shifts in microbial composition and diversity. This study aimed to investigate the compositional dynamics of the oral microbiota during the early stages of gingival inflammation, focusing on changes across multiple oral niches and their relationship to clinical outcomes.

**Methods:**

We conducted an experimental gingivitis intervention study with 41 healthy volunteers. After a two-week baseline period, participants refrained from oral hygiene for two weeks to induce gingivitis, followed by a one-week resolution phase with resumed oral hygiene. Clinical parameters, including plaque and bleeding scores, were monitored at seven time points. Samples from saliva and five oral niches (tongue, keratinized gingiva, supragingival, subgingival, and interproximal dental plaque) were collected and analyzed using 16S rRNA gene sequencing. Multivariate statistical analyses were applied to evaluate microbial dynamics and their associations with clinical outcomes.

**Results:**

The study revealed pronounced microbial changes, particularly in supragingival plaque, where *Leptotrichia* and *Prevotella* increased while *Streptococcus* decreased. Alpha diversity significantly increased in supragingival plaque, tongue, and saliva during gingivitis induction, highlighting shifts in microbial complexity. Clinical correlations indicated that plaque presence was primarily associated with bacterial load, while gingival bleeding was driven by compositional changes in supragingival plaque and tongue biofilms. These findings suggest that microbial density and composition independently contribute to gingivitis markers.

**Conclusion:**

This study concludes that occurrence of dental plaque and gingival bleeding are independent clinical parameters, linked to bacterial load and composition, respectively. Interactions between multiple niches, especially the tongue, influence clinical outcomes, highlighting a complex, nonlinear dynamic behavior in the oral microbiota. These findings suggest intricate ecological interactions that may approach tipping points, advancing understanding of microbial dynamics during gingival inflammation and informing future strategies for managing gingivitis.

## Introduction

Oral health is a vital aspect of overall health and quality of life, yet oral diseases remain a prevalent and significant global public health challenge (Kassebaum et al., 2017). The human mouth hosts a complex and dynamic environment, with diverse ecological niches formed by both hard tissues (teeth) and soft tissues such as the gingiva and tongue. These niches are further influenced by anatomical variations, including proximity to salivary ducts and exposure to mechanical forces, resulting in distinct microbial communities. These communities, consisting of bacteria, fungi, archaea, viruses, and protozoa, are among the most diverse in the human body, second only to the colon (The Human Microbiome Project Consortium, 2012). These microorganisms often form biofilms, such as dental plaque, where bacteria dominate and include both aerobic and anaerobic species. Obligate anaerobes thrive in oxygen-deprived niches, such as the gingival sulcus, where bacterial genera such as *Porphyromonas, Tannerella, Prevotella, Fusobacterium*, and *Treponema*, are prevalent (Dewhirst et al., 2010). These microorganisms extract energy from many host-derived nutrients, such as glycoproteins and other salivary components (Kilian et al., 2016).

Microbial interactions in the oral cavity support a commensal relationship with the host, promoting mutual benefits through physical attachment, protection from abrasive forces, and metabolic interdependencies (Takeshita et al., 2016; Lamont et al., 2018). Factors such as food intake, oral hygiene routines, and the biochemical environment shape the composition and activity of these microbial communities (Wade, 2013). A disruption of the homeostasis in the oral ecosystem can lead to shifts in microbial composition, often described as dysbiosis, characterized by increased proportions of species with pathogenic potential, such as *Porphyromonas gingivalis*, *Treponema denticola*, *Fusobacterium nucleatum*, and *Prevotella* spp (Sultan et al., 2018). In this way, microorganisms normally residing in the oral cavity can contribute to different diseases, such as periodontitis, caries, root canal infections and others. There is also evidence, both in animal model studies and in humans, that oral dysbiosis may be associated with systemic inflammation and to diseases such as diabetes, pneumonia, atherosclerosis, inflammatory bowel disease and others (Arimatsu et al., 2014; Kramer and Genco, 2017; Kumar, 2017).

Previous studies investigating the oral microbiota found that a specific microbiome is associated with oral health (Takeshita et al., 2016) and that certain microbial signatures can indicate an early shift towards dysbiosis (Zaura et al., 2017). One study induced experimental gingivitis in healthy individuals to characterize the composition of the microbial communities in plaque during the transition from health to disease and found that the transition from periodontal health to gingivitis involves significant shifts in the bacterial community structure, with an increase in diversity and specific gingivitis-associated taxa such as *Fusobacterium nucleatum*, *Prevotella oulorum*, and others, highlighting potential targets for periodontal disease prevention and therapy (Kistler et al., 2013).

Here, we used a similar approach to induce gingivitis and studied its effect on clinical parameters and on the dynamic changes occurring in bacterial community composition across multiple oral niches. To achieve this, we conducted an experimental gingivitis intervention study that measured gingival bleeding, dental plaque, and microbiota composition across different oral ecological niches. The study explored how bacterial groups were distributed across niches and how their composition changed over time during the experimental period. Additionally, we investigated the associations between specific bacterial taxa and clinical markers such as gingival bleeding and plaque indices. Rather than establishing causality, our aim was to examine how biofilm buildup and composition relate to clinical changes, recognizing inter-individual variability in response. This approach provides a comprehensive understanding of the relationships between microbial dynamics and clinical outcomes during gingivitis, offering insights into niche-specific contributions to oral health and potential avenues for targeted therapeutic strategies.

## Materials and methods

### Intervention and sample collection

**Figure 1:**
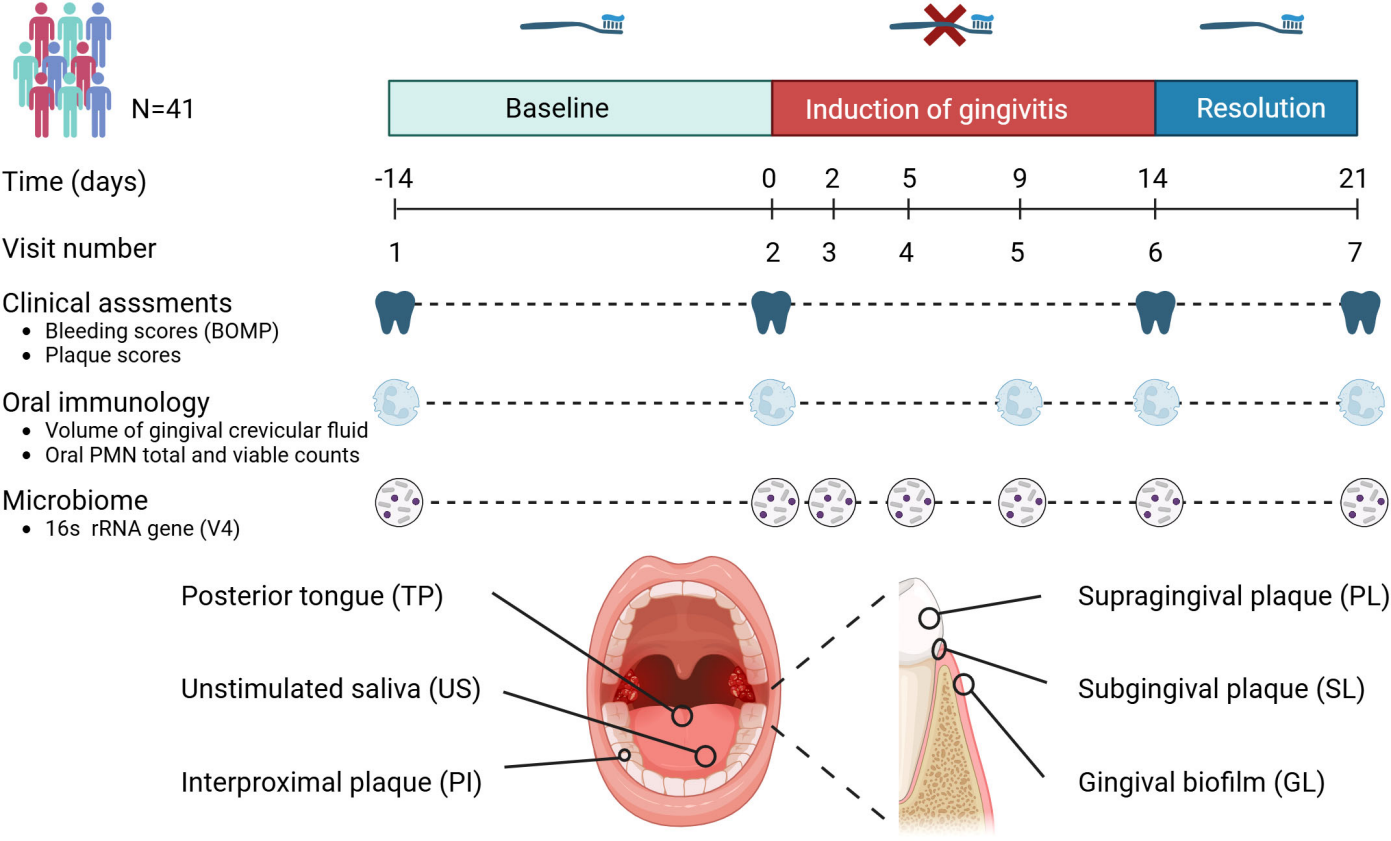
Timeline and study set-up. This longitudinal study involved 41 volunteers. The study set-up consisted of a two-week baseline period during which volunteers brushed teeth with a standardized toothbrush and toothpaste. After this period, volunteers refrained from oral hygiene for two weeks (induction of gingivitis period), followed by a one-week period where volunteers reinstated their oral hygiene routines. Assessments included clinical, inflammatory and immunological parameters as well as microbiome assessments of six different niches within the oral cavity.

The study included 41 individuals between 18 and 45 years of age (15 men and 26 women). Study details are described in van der Veen et al. (van der Veen et al., 2016). The study participants were systemically healthy volunteers, as determined through a medical questionnaire. Exclusion criteria included the use of antibiotics within the three months preceding the study and smoking. Non-smokers were defined as individuals who smoked fewer than one cigarette per day for at least one year. Experimental gingivitis was induced by eliminating all oral hygiene for two weeks (no brushing or flossing, no use of toothpaste or any mouth rinses). The study consisted of three time periods: the two weeks preceding the intervention (“baseline”), the two intervention weeks (“experimental gingivitis”) and the week following it (“resolution”) (**Figure 1**). At the start of the resolution phase, all participants received a standardized manual toothbrush, fluoride toothpaste, and detailed instructions to resume tooth brushing. No prophylactic cleaning was performed at the start of the experimental gingivitis period. Throughout the study, samples were collected, and clinical parameters were measured during seven visits: Visit 1 marked the start of the baseline period (day -14); Visit 2 served as both the conclusion of the baseline period and the beginning of the intervention (day 0); Visits 3 to 6 took place during the intervention phase (days 2, 5, 9, and 14); and Visit 7 marked the end of the resolution phase (day 21). Six different oral samples were taken at every visit: supragingival plaque (PL), subgingival plaque (SL), interproximal plaque (PI), lower jaw gingiva (GL), unstimulated saliva (US) and posterior part of tongue dorsum (TP) (**Figure 1**). As the collection of plaque samples could potentially interfere with plaque maturation during experimental gingivitis, a sampling scheme was defined in which at each visit either the distal or mesial buccal (vestibular) surface of the first molar of the lower left or right quadrant would be sampled. Interproximal plaque was collected between the first and second molar of all four quadrants. These samples were collected from opposing quadrants of those used for dental plaque assessment (**Table 1**). All sampling occurred between 09:00 and 12:00 A.M., with participants instructed to avoid eating or drinking (except water) for two hours before each visit.

**Table 1:** Sampling scheme for dental plaque samples.

	Visit						
	1	2	3	4	5	6	7
	Day -14	Day 0	Day 2	Day 5	Day 9	Day 14	Day 21
Sample site	Tooth code						
Supragingival plaque (PL)	3.6/4.6 MV	3.6/4.6 MV	3.6/4.6 DV	3.7/4.7 MV	3.7/4.7 DV	3.6/4.6 MV	3.6/4.6 DV
Subgingival plaque (SL)	3.6/4.6 ML	3.6/4.6 ML	3.6/4.6 DL	3.7/4.7 ML	3.7/4.7 DL	3.6/4.6 ML	3.6/4.6 DL
Interproximal sample (Pi)	1.6|1.7 upper	1.6|1.7 upper	4.6|4.7 lower	1.5|1.6 upper	4.5|4.6 lower	1.6|1.7 upper	1.6|1.7 upper

Plaque samples were collected at different teeth [indicated by the ISO 3950:2016 standard numbering (ISO, 2016)]. At each visit, sampling of supragingival and subgingival plaque was performed at either the distal (D) or mesial (M) buccal (vestibular (V)) surface of the first molar of the lower left or right quadrant. Interproximal plaque was collected between the first and second molar (6/7) or premolar and molar (5/6) in the upper jaw, opposing the quadrant for dental plaque assessment. Quadrant numbers are denoted as follows: 1 (Upper Right), 2 (Upper Left), 3 (Lower Left), and 4 (Lower Right).

Unstimulated saliva was collected following the previously described procedures (Zaura et al., 2017). The tongue dorsum was sampled by stroking the posterior part of the dorsum of the tongue four times in a longitudinal direction with a microbrush (Microbrush International, Grafton, USA). The tip of the microbrush was clipped off in an Eppendorf tube prefilled with 50 µl RNAlater (ThermoFisher Waltham, MA, USA). Samples from the attached gingival tissue were collected using a sterile, soft-tipped microbrush, gently rotated against the tissue surface after which the tip of the microbrush was clipped off in an Eppendorf tube prefilled with 50 µl RNAlater. The anatomic location for collecting keratinized gingiva corresponds to the attached gingival tissue adjacent to the teeth where supragingival plaque was sampled, as outlined in **Table 1**. Supragingival plaque was collected from the vestibular (buccal) molar surface with a sterile Teflon spatula (KerrHawe, Bioggio, Switzerland). Subgingival plaque was sampled using a sterile curette. Plaque samples were transferred to an Eppendorf tube prefilled with 50 µl RNAlater (Thermo Fisher Scientific, Ambion, Waltham, MA, USA), spun down for 1 minute at 14,000 rpm and stored on ice for maximal 2 hours and stored at -80°C. Interproximal plaque was collected using unwaxed dental floss. Plaque was transferred to an Eppendorf tube prefilled with RNAlater by passing the floss thread through a slit made in the lid of the tube. After a quick spin in the centrifuge, samples were stored on ice for maximal two hours before transfer to -80°C.

Gingival bleeding and plaque levels were measured during study visits 1, 2, 6, and 7 to evaluate temporal changes. Gingival bleeding was assessed by marginal probing (BOMP), involving running a probe along the marginal gingiva, at an angle of 60° to the longitudinal axis of the tooth, scored no bleeding (0); pin-prick bleeding (1); excessive bleeding (2) at 6 sites of each tooth (Van der Weijden et al., 1994). The amount of plaque was assessed clinically by scoring 6 sites of each tooth on a four-point scale (0-3) using a modified Silness & Löe Index (Silness and Loe, 1964). Plaque and bleeding scores were recorded for two quadrants opposite to the sites where plaque biofilm samples were collected.

The percentage of plaque was determined by categorizing plaque scores as either absent or present. Plaque scores of 1, 2, and 3 were combined into a single score of 1. This combined score was then divided by the total number of assessed sites and multiplied by 100 to obtain a percentage score. In a similar approach, BOMP percentages were calculated, following dichotomization of the BOMP scores in presence (score 1 and 2) or absence (score 0) of bleeding.

### Oral immunology

For enumeration of Oral Polymorphonuclear Neutrophils (oPMNs), oral rinses were collected from each participant at day -14, day 0, day 9, 14, and day 21 between 09:00 and 12:00 A.M. The collection process adhered to a previously established protocol (Rijkschroeff et al., 2016; Rijkschroeff et al., 2020) and was adapted from foundational isolation methodologies described by Ashkenazi and Dennison (1989), and al-Essa et al. (al-Essa et al., 1994). Participants were instructed to perform five consecutive oral rinses with a 5 mL of sterile sodium chloride solution (0.9% NaCl) per rinse, with each rinse lasting 30 seconds and a 3½ minute break between rinses. To minimize contamination by pharyngeal or epithelial cells, participants were explicitly instructed not to gargle or clear their throat during the sampling. The pooled samples obtained from these rinses were stored on ice in a 50 mL centrifuge tube (SigmaAldrich, Zwijndrecht, The Netherlands) until the collection process was complete. To enrich and purify oPMNs, the oral rinse samples were passed through sequential, nylon mesh filters (Cytek Biosciences, Inc., Fremont, CA, USA) with decreasing pore sizes of 70 µm, 30 µm, and 10 µm, consistent with the filtration strategy proposed by al-Essa et al., which effectively removes cellular debris and larger epithelial cells while retaining viable neutrophils. After filtration, the samples were centrifuged at 500 g for 10 minutes at 4°C. The supernatant was discarded, and the pelleted cells were resuspended in phosphate-buffered saline (Gibco, Paisley, United Kingdom) while being kept on ice for subsequent cell quantification. The quantification of cells was carried out using a Muse Cell Analyzer (Merck Millipore) with the Counts and Viability kit, following the manufacturer’s instructions. Gingival Crevicular Fluid (GCF) volume was determined for each participant at day -14, day 0, day 9, 14, and day 21. For GCF volume analysis, the area to be sampled was isolated with cotton rolls, and the teeth were gently air‐dried for 5 s. The PerioPaper collection strips (Oraflow Inc., Plainview, NY, USA) were placed for 30 s in the sulcus. The volume was determined using an electronic transducer (Periotron 8000) in accordance with the manufacturer’s recommendations (Oraflow).

### DNA isolation, bacterial load, and 16S rRNA gene amplicon sequencing

DNA was isolated from the samples using the method described by Zaura et al (Zaura et al., 2017). Samples were mixed with 300 µl lysis buffer (Agowa, Berlin, Germany), 500 µl phenol saturated with tris-HCl (pH 8.0) and 500 µl zirconium beads (0.1 mm; BioSpec Products, Bartlesville, OK, USA), and shaken in a bead beater for 3 min at 2800 oscillations/min. Next, DNA was purified using magnetic beads (Agowa, Berlin, Germany) and used for amplicon sequencing. Bacterial load of all samples was determined by quantitative PCR using primers S001 (5’-GTT CGT ACT CCC CAG GCG G-3’), S002 (5’-CGA AAG CGT GGG GAG CAA A-3’) and probe S003 (5’-FAM-ATT AGA TAC CCT GGT AGT CCA-MGB-3’). qPCR was performed in RT PCR master mix (Diagenode, Seraing, Belgium) on an Applied Biosystems 7500 RT PCR system during 45 cycles of with a denaturation step at 95.0°C for 15 s and an annealing/elongation step at 60.0°C for 1 min. The V4 hypervariable region of the 16S rRNA gene was targeted using primers F515 (5′- GTG CCA GCM GCC GCG GTA A -3′) and R806 (5′- GGA CTA CHV GGG TWT CTA AT -3′). The primers included Illumina adapters and a unique 8-nucleotide sample index sequence key (Kozich et al., 2013). PCR was performed using the Phusion Hot Start II High Fidelity PCR Master Mix (Thermo Scientific, Waltham, MA, USA) with 100 pg template. The following amplification program was used: initial denaturation at 98°C for 30s; 30 cycles of 98°C for 10s, 55°C for 30s and 72°C for 30s; and final elongation at 72°C for 5 min. The amplicon libraries were pooled in equimolar amounts and purified using the QIAquick Gel Extraction Kit (Qiagen, Valencia, CA, USA). Amplicon quality and size were analyzed on a Fragment Analyzer (Advanced Analytical Technologies Inc., Ankeny, IA, USA). The pooled amplicon library was quantified using the Quant-iT™ PicoGreen^®^ dsDNA Assay Kit (Thermo Fisher Scientific). Amplicon sequencing was performed on the Illumina MiSeq platform (Illumina Technologies, San Diego, CA, USA) using 2 x 200 cycle paired-end settings. In each run, a mock control, three independent saliva samples (US2341, US2342, US2385) and a pooled saliva sample was included. Technical variation was evaluated by calculating the coefficient of variation (CV) for the mean reads per sample across 30 sequencing runs (**Supplementary Figure S1**).

### Sequencing data processing

First, low-quality regions were trimmed using Btrim (Kong, 2011) with a sliding window size of 5 nt and average quality score of 25. Next, the paired-end reads were processed using Mothur v.1.36.1 (Schloss et al., 2009) to merge and filter the reads of the sequences as follows. Unique sequences were filtered by length (range: 243 – 263), with no ambiguous bases allowed. Unique, filtered sequences were aligned to the bacterial SILVA SEED (version v.123) reference database (Schloss et al., 2009). Chimeric reads were identified (*de novo*) and removed using UCHIME (Edgar et al., 2011). Sequences occurring less than (<) 10 times in the entire dataset were removed. Subsampling was performed to normalize the number of reads per sample to 10 000. Minimum Entropy Decomposition (MED) (version 2.1) (Eren et al., 2015) was used to partition the amplicon sequences into MED nodes, which were taxonomically classified using the HOMD database version 13.2 (Chen et al., 2010) and the RDP naïve bayes classifier from Mothur (1000 min confidence MED uses entropy threshold (parameter MED200) to filter the sequences by variability in nucleotide positions and thereby decomposing nodes until convergence is reached.

All analyses of the microbiome dataset were performed using R version 4.2.2 (R core team, 2022). All figures were composed using the *ggplot2* package version 3.4.1 (Wickham, 2016).

### MED nodes data processing

The MED node count data matrix was filtered to include only those taxa that contribute to the first 99% of the combined microbial communities (Fluitman et al., 2021), as described below. This step was not performed for the dataset that was used for alpha-diversity analysis. Each row (representing one sample) of the count table was normalized to the sample’s sum. Each column (representing relative abundances of one taxon) was summed up and sums were sorted in decreasing order. Cumulative sums of the sorted column sums were divided by the total of the column sums and taxa represented by values above 0.99 were marked for removal. Any taxon with values smaller than 0.99 (making up the first 99% of the total communities) were kept in the original count data table for the following analyses. This procedure removes low count taxa from the dataset.

For the PCA, distance and classification analysis, the data was transformed using the centered log-ratio (CLR) transformation, to account for the compositional nature of 16S rRNA gene sequencing data. This was done using the *compositions* package, version 2.0-5 (Greenacre, 2021).

### Principal component analysis and beta distance analysis

Principal component analysis of the CLR transformed MED count data was performed using the *pcaMethods* package, version 1.90.0 (Greenacre, 2021).

Distance analysis was conducted using Aitchison distance on the count data. Specifically, the Aitchison distance in relation to visit 2 was calculated for each visit, within each subject, within each niche. The resulting distances were then subjected to analysis using a linear mixed-effects model, as described below.

### Linear mixed-effects models on MED data

Regression and statistical testing on the MED count data was in all cases performed using linear mixed-effects models from the *dream* package, version 1.23.0 with the *variancePartition* extension (Hoffman and SChadt, 2016; Hoffman and Roussos, 2021). This combination of packages provides functions for differential abundance testing using negative binomial linear mixed models for repeated measures count data. To ensure homogeneity of variances (homoscedasticity) of the model residuals, the gingival bleeding and plaque index data underwent square-root and square transformations, respectively. P-values were adjusted using the Benjamini-Hochberg procedure for multiple comparisons, per niche, for bleeding and plaque separately (Benjamini and Hochberg, 1995).

### Linear mixed-effects models for other data

For variables that were not part of the microbiome data, statistical models were created using the *lme4* package (Bates et al., 2015). The variables representing the number of oPMN cells, number of viable oPMN cells, and the bacterial load data were log-transformed before model fitting, while gingival bleeding data was log-transformed and fitted using weighted least-squares with sample weight inverse-reciprocal to the response variable. These transformations were applied to ensure normality and homoscedasticity of the model residuals. Samples with standardized residuals at a distance greater than 3 standard deviations from 0 were excluded as statistical outliers.

After model fitting, hypothesis testing was performed using the *emmeans* package (Lenth, 2022). In cases where the model response variable was transformed, the model was back-transformed to the original response scale before hypothesis testing. No multiple testing correction was applied, and P-values less than 0.05 were considered statistically significant.

### Regression model

A multinomial Elastic et classification model was created using the *glmnet* package to identify important MED nodes in supragingival plaque (PL) (Friedman et al., 2010). The model was trained solely on data from the PL niche that was pre-processed by mean-centering and scaling to unit variance. Because of the high similarity in microbial composition of the visits from the brushing phase, data from visit 1 & 7 were excluded from this model. The Elastic et mixing parameter α was fixed at 1, thus LASSO penalties were enforced during model fitting. To optimize the regularization parameter λ, a 10-fold cross-validation procedure was used to minimize the classification error. The largest value of λ was selected such that the model classification error at that value was within 1 standard error of the minimum classification error.

Model performance measures were calculated using the *caret* package at the selected value of λ (Kuhn, 2008). To determine the relative importance of each MED node in the classification procedure at each time point, the resulting model coefficients were multiplied by the standard deviation of the corresponding input feature and the absolute values of these standardized coefficients were taken. This yielded a value for each taxon that reflects its relative weight in the classification procedure.

### Correlation network

The *rstatix* package was used to compute pairwise Spearman correlations (ρ) across all clinical (plaque, bleeding), immunological (cell counts, viability, GCF volume), bacterial load, and Aitchison distance metrics (Kassambara, 2023). Aitchison distances were calculated for each visit relative to the baseline (Visit 2), resulting in multiple distance values per participant over time (i.e., Visits 3, 4, 5, 6, and 7). Correlations were computed using pairwise complete observations, meaning that each correlation was based on the subset of time points where data for both variables were present for the participants. Only correlation pairs with p ≤ 0.05 were retained, and the absolute value of ρ was used as an edge weight in the network. This approach effectively integrates multiple time points, capturing the longitudinal structure of associations. Node positions in the network were determined using a force-directed layout, with edge thickness corresponding to |ρ|, and node colors indicating variable categories (clinical, immune, microbial load, microbial distance).

## Results

### Effect of experimental gingivitis on clinical and immunological parameters

**Figure 2:**
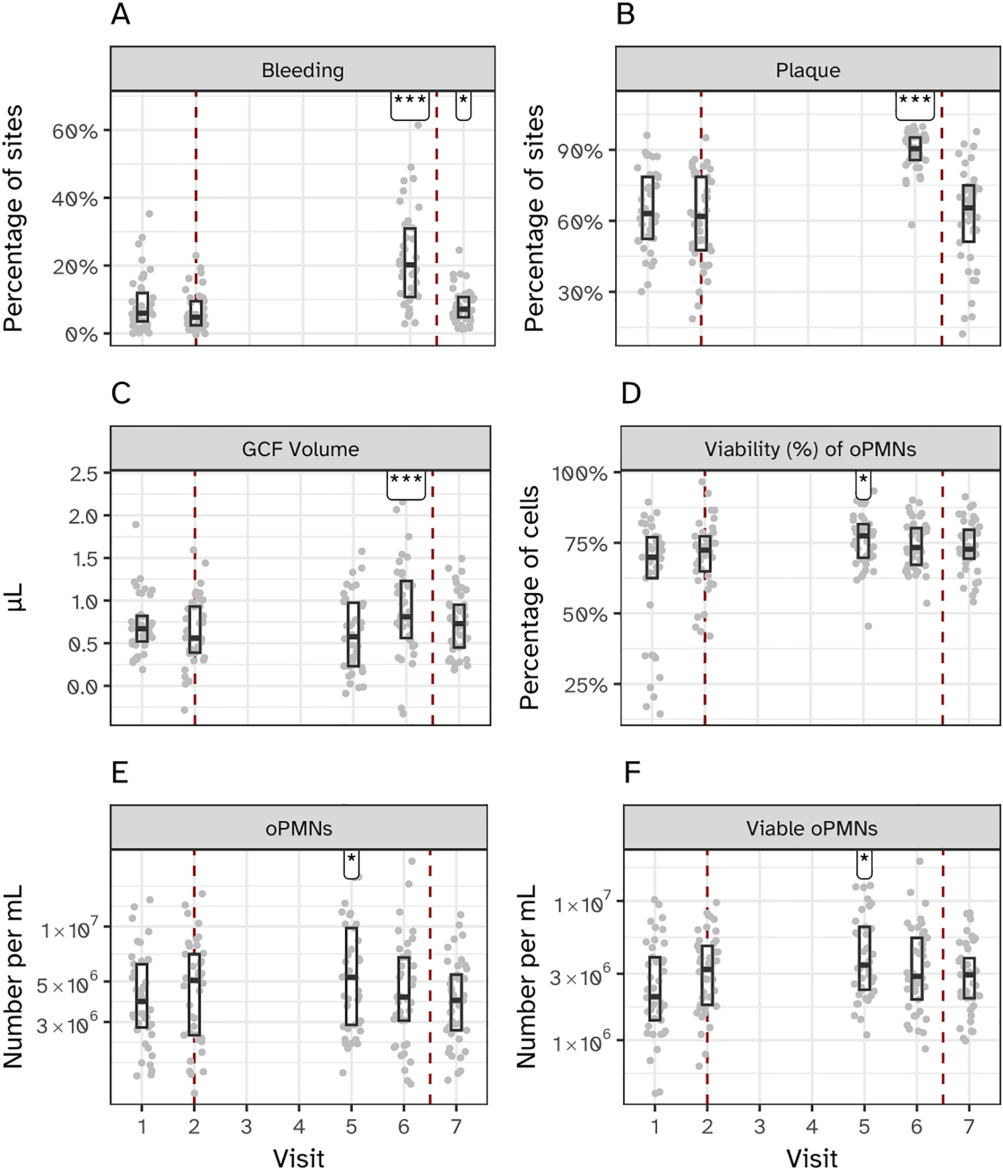
Clinical and immunological effects of experimental gingivitis. **(A)** Percentage of bleeding sites, and **(B)** percentage of plaque-covered sites relative to all examined sites. Measurements for panels A and B were taken before the induction of gingivitis (visits 1 and 2; days -14 and 0), at the end of the experimental period (visit 6; day 14), and one week post-experiment (visit 7; day 21). **(C)** Volume of gingival crevicular fluid (GCF). **(D–F)** Percentage of viable oral polymorphonuclear neutrophils (oPMNs), total oPMN count, and viable oPMN count, respectively. Measurements for **(C–F)** were taken before gingivitis induction (visits 1 and 2), during the experimental period (visit 5; day 9), at its conclusion (visit 6; day 14), and one week post-experiment (visit 7; day 21). Significant differences compared to visit 2 are indicated (*P < 0.05, ***P < 0.001).

Forty-one volunteers completed the experimental gingivitis intervention. Clinical parameters (occurrence of percentage of gingival sites with bleeding upon marginal probing (BOMP) and occurrence of dental plaque) were measured at two visits before initiation of the two weeks experimental gingivitis period (visit 1 and 2), upon completion (visit 6) and after one week of brushing (visit 7) (**Figure 1**). The intervention (i.e. abstention from oral hygiene) resulted in a significant increase in supragingival plaque and gingival bleeding (**Figure 2**), as compared to the end of the baseline and start of the intervention (visit 2). Upon re-initiation of brushing, occurrence of dental plaque returned to baseline; the percentage of bleeding sites also decreased compared to Visit 6 but remained significantly higher than at visit 2 (baseline).

The volume of gingival crevicular fluid (GCF), a clear serum-like fluid naturally present in the gingival sulcus, was measured to assess the gingival inflammatory response (**Figure 2C**). GCF volume significantly increased by day 14 (Visit 6) of the experimental gingivitis phase, with no significant change observed at day 9 (Visit 5). The total number of oral polymorphonuclear neutrophils (oPMNs), a key component of the innate immune response that migrate to the gingival crevice during inflammation, was also quantified (**Figures 2D–F**). oPMN counts peaked at day 9 and decreased by day 14, while the percentage of viable oPMNs initially increased during the gingivitis phase but returned to baseline levels by the final visit. Due to the absence of intermediate sampling points, earlier peaks in oPMN counts cannot be excluded. These measurements highlight distinct temporal changes in GCF volume and oPMN dynamics over the course of the experimental gingivitis phase.

### Effect of experimental gingivitis on the oral microbiota

**Figure 3:**
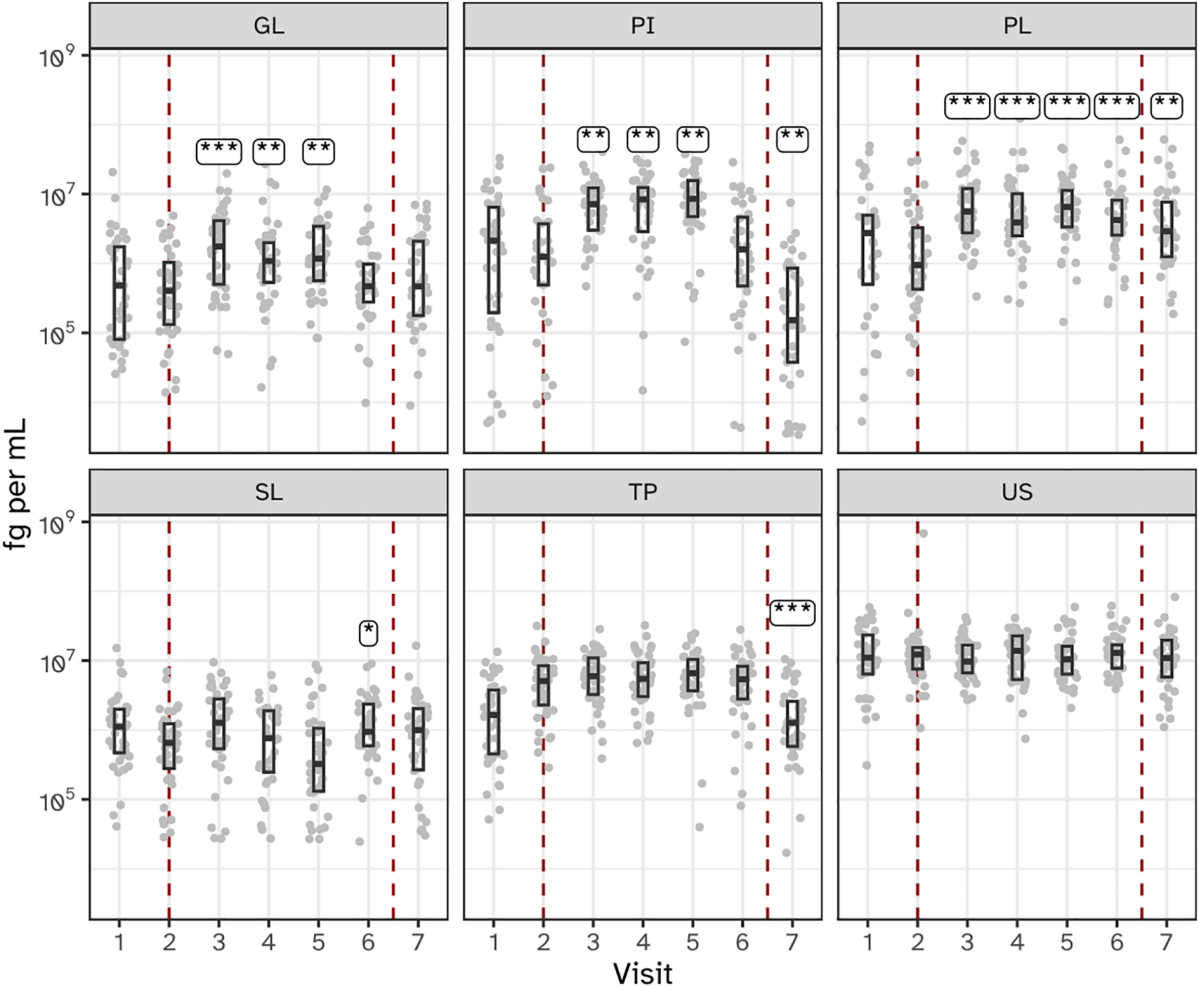
Bacterial load, as determined by 16S ribosomal gene abundance (qPCR) in the six niches as assessed as quantity of femtograms bacterial genomic DNA per mL, throughout the study. Annotations indicate a significant difference versus visit 2 (***P < 0.001; **P < 0.01; *P < 0.05). The microbiota of six oral niches sampled were lower jaw gingiva (GL), interproximal plaque (PI), supragingival plaque (PL), subgingival plaque (SL), posterior tongue (TP) and unstimulated saliva (US). Visits 1 and 2 - baseline, visit 3 - day 2, visit 4 - day 5, visit 5 - day 9 and visit 6 - day 14 of experimental gingivitis, visit 7 - day 7 of the resolution phase.

Bacterial load, as measured by 16S rRNA gene qPCR, varied significantly across the six sampled niches during the experimental gingivitis phase. Supragingival plaque (PL) showed a sustained increase in bacterial load throughout the experimental gingivitis phase and remained increased at the end of the resolution phase (visit 7), indicating persistent changes even after oral hygiene was reinstated (**Figure 3**). In contrast, interproximal plaque (PI) and gingival biofilms (GL) displayed transient increases in bacterial load, peaking early during the gingivitis phase (day 5–9), before returning to baseline levels by the end of the phase (day 14). Subgingival plaque (SL), posterior tongue (TP), and unstimulated saliva (US) showed minimal or no significant changes in bacterial load during the experimental period. These findings indicate that the microbial response to disrupted oral hygiene varies by niche, with supragingival plaque being the most significantly affected, followed by interproximal plaque and gingival biofilms.

### Overall sequencing results

By sequencing the V4 hypervariable region of the 16S rRNA gene, the bacterial composition was determined in all 1722 samples, yielding 128,617,955 reads. Sequencing depth varied across the six sample types, with a mean read count ranging from 61,142.06 ± 35,800.14 (US) to 90,733.45 ± 44,229.68 (PI), and an overall mean of 77,109.09 ± 44,713.93 reads per sample. The number of samples per category ranged from 262 (PI) to 287 (TP and US), with total read counts per sample type spanning 17,547,770 (US) to 24,663,513 (PL). Across the six niches, 2358 microbial sequence variants (Minimum Entropy Decomposition (MED) nodes) were identified. After abundance filtering, 723 MEDs were included in the analysis, corresponding to 93.5% of the reads. 4.0% of the reads were not assigned to a MED node and 2.5% reads were excluded after filtering.

### Induction of gingivitis coincides with increased alpha diversity

**Figure 4:**
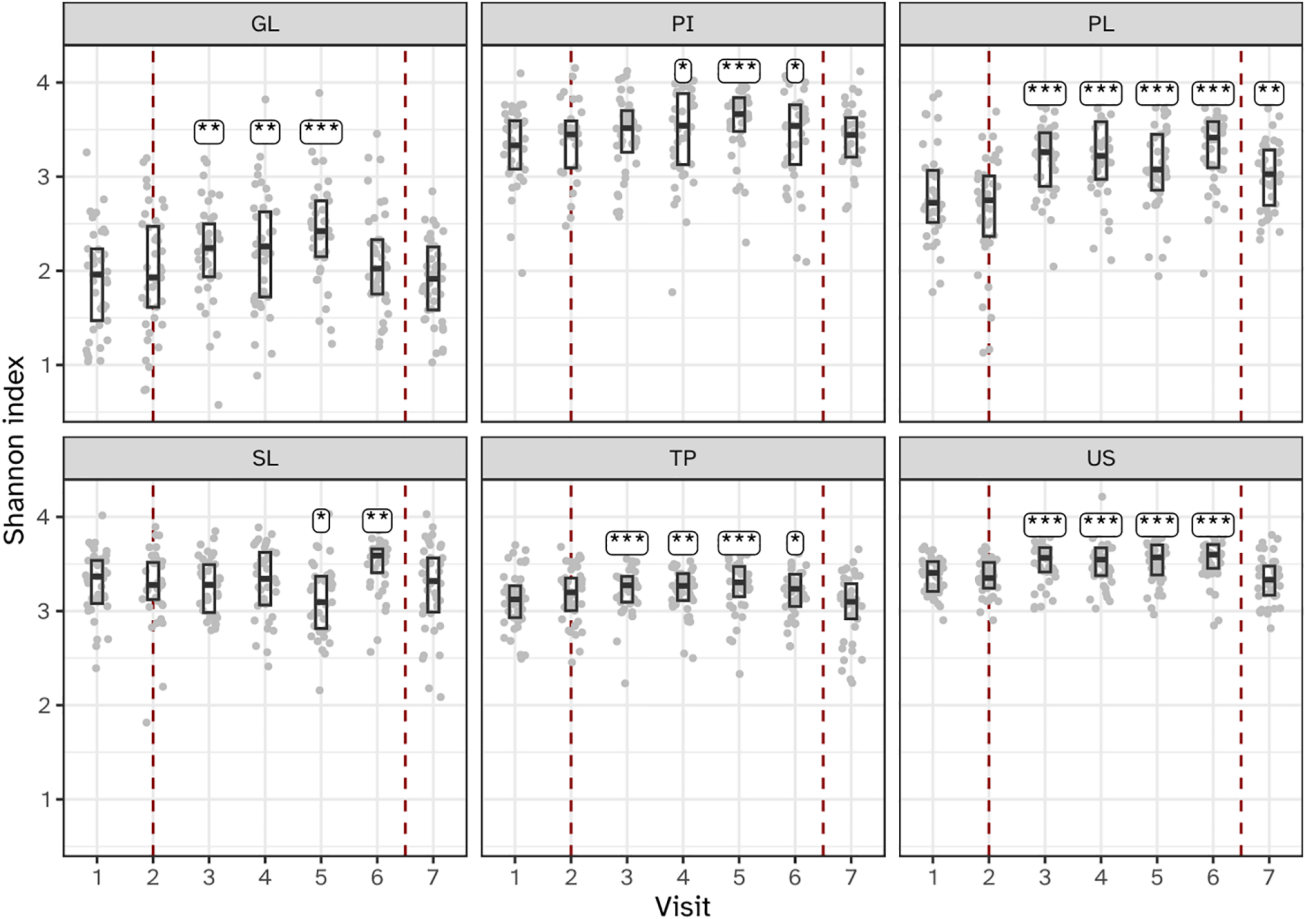
Boxplots showing microbial alpha diversity, as determined by the Shannon index of the six niches throughout the study. Annotations indicate a significant difference versus visit 2 (***P < 0.001; **P < 0.01; *P < 0.05). The microbiota of six oral niches sampled were: lower jaw gingiva (GL), interproximal plaque (PI), supragingival plaque (PL), subgingival plaque (SL), posterior tongue (TP) and unstimulated saliva (US). Visits 1 and 2 - baseline, visit 3 - day 2, visit 4 - day 5, visit 5 - day 9 and visit 6 - day 14 of experimental gingivitis, visit 7 - day 7 of the resolution phase.

The alpha diversity of biofilms from all six niches increased during experimental gingivitis at least at one time point compared to baseline (**Figure 4**; **Supplementary Figures S2**, **S3**). Most profound increase in the number of MEDs observed as well as in the Shannon diversity indexes were observed for supragingival plaque (PL), tongue (TP) and saliva (US) where a significant (P<0.05) increase in diversity was observed at all timepoints during experimental gingivitis compared to baseline visit 2. For other niches, the increase in alpha diversity was limited to only a few timepoints. For interproximal plaque (IP), a significant increase in diversity was detected between visits 4 and 6 (days 5 – 14). The subgingival plaque (SL) microbiota showed a significant increase in alpha diversity at visit 5 (day 9) and 6 (day 14). For the keratinized gingiva (GL), a significant increase in alpha diversity was observed for samples collected at visits 3 (day 2), 4 (day 5), and 5 (day 9), but not at 6 (day 14).

### Changes in microbial composition – beta diversity

**Figure 5:**
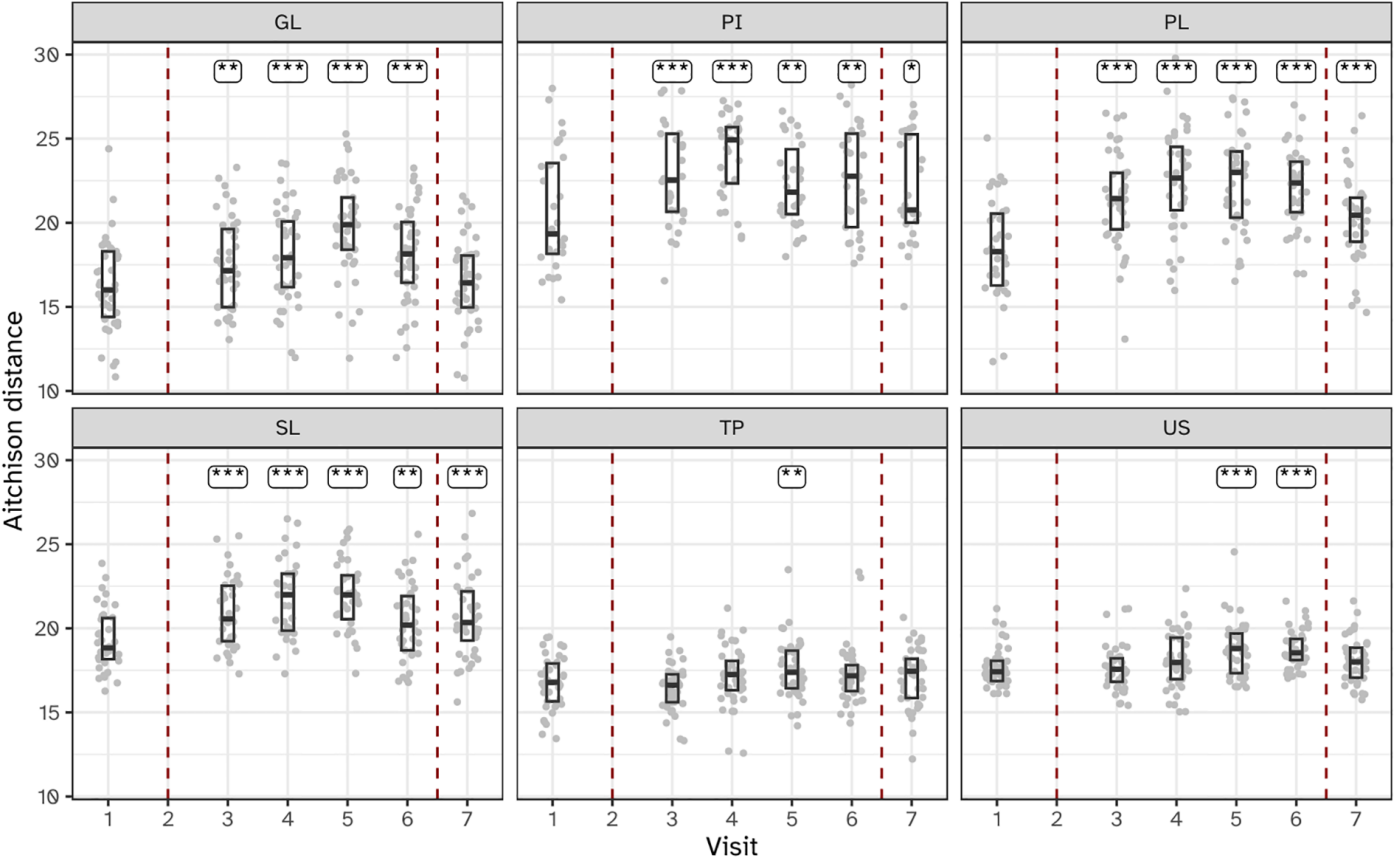
Boxplots showing within-subject Aitchison distance from visit 2 for the six niches throughout the study. Annotations indicate a significant difference versus visit 2 (***P < 0.001; **P < 0.01; *P < 0.05). The microbiota of six oral niches sampled were: lower jaw gingiva (GL), interproximal plaque (PI), supragingival plaque (PL), subgingival plaque (SL), posterior tongue (TP) and unstimulated saliva (US). Visits 1 and 2 - baseline, visit 3 - day 2, visit 4 - day 5, visit 5 - day 9 and visit 6 - day 14 of experimental gingivitis, visit 7 - day 7 of the resolution phase.

The within-subject beta diversity distances were calculated for all niches and timepoints compared to baseline (**Figure 5**). As expected, no significant (P<0.05) changes occurred in microbiota composition in the two visits preceding the start of the experimental gingivitis study. During the experimental gingivitis period, the microbiota of all microbial samples showed a significant increase in beta diversity compared to baseline at one or more timepoints. Progression of experimental gingivitis did not coincide with a continued increase in beta diversity. After an increase in beta distance during the first 5 to 9 days for GL, PI, PL and SL, the distance reduced at later time points. Such behavior was also observed for the tongue biofilm, where a significant increase in comparison to the baseline, was observed only at visit 5 (day 9) but not at any other time point. One week after re-initiation of tooth brushing, the microbiota composition of half of the niches returned to baseline levels, except for the supragingival (PL), interproximal (PI), and subgingival plaque (SL) samples.

### Niche-dependent differences in temporal effects on microbiota composition

**Figure 6:**
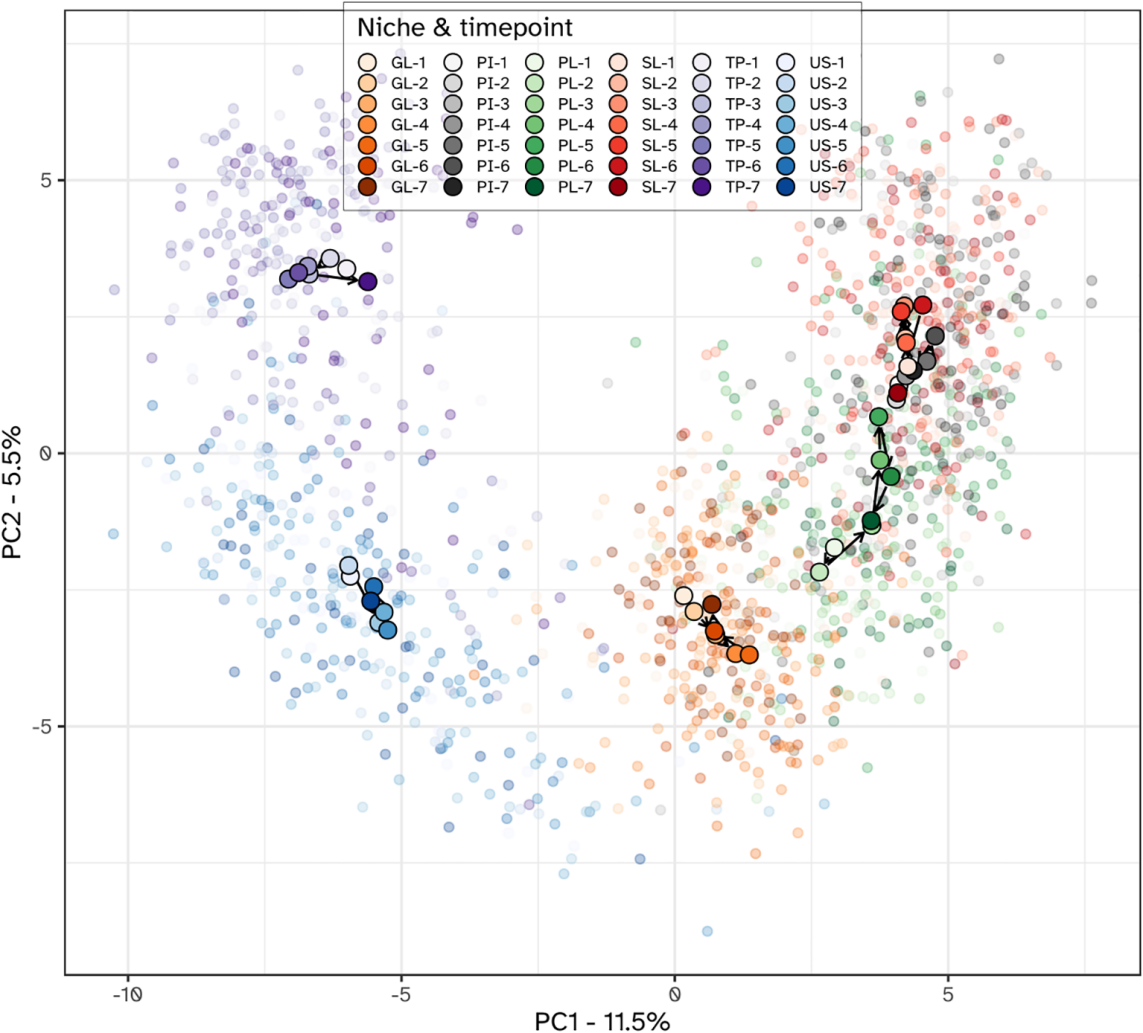
PCA plot of the microbial communities from all niches. Individual samples are shown using translucent dots. The centroid value for all subjects within each niche-time combination is shown with a larger filled dot. Color hues correspond to different niches, with the tint varying from light to dark, corresponding with the visits. Within each niche, subsequent visits are connected with arrows, starting from the first visit and ending with the last. Niches were the lower jaw gingiva (GL), interproximal plaque (PI), supragingival plaque (PL), subgingival plaque (SL), posterior tongue (TP) and unstimulated saliva (US). The timepoints were: 1 and 2 - baseline; 3 - day 2, 4 - day 5, 5 - day 9 and 6 - day 14 of experimental gingivitis; 7 - day 7 of the resolution phase.

Principal component analysis (PCA) unveiled distinct stratification of microbiota composition based on ecological niches, alongside niche-specific alterations observed during the intervention (**Figure 6**). The first PCA axis (PC1) discriminated between dental biofilms (supragingival plaque, subgingival plaque and interproximal plaque) and those from the tongue and saliva. The gingiva samples localized between the hard and soft tissue biofilms in the ordination plots. Examining the PCA loadings driving the niche-dependent differences suggested that compared to dental plaque samples, *Prevotella*, *Veillonella* and *Actinomyces* species were more predominant in tongue and saliva samples. The tongue biofilm was enriched with *Leptotrichia* species compared to other niches. Gingival biofilm samples were enriched with *Haemophilus*, *Streptococcus* and *Granulicatella* species.

**Figure 7:**
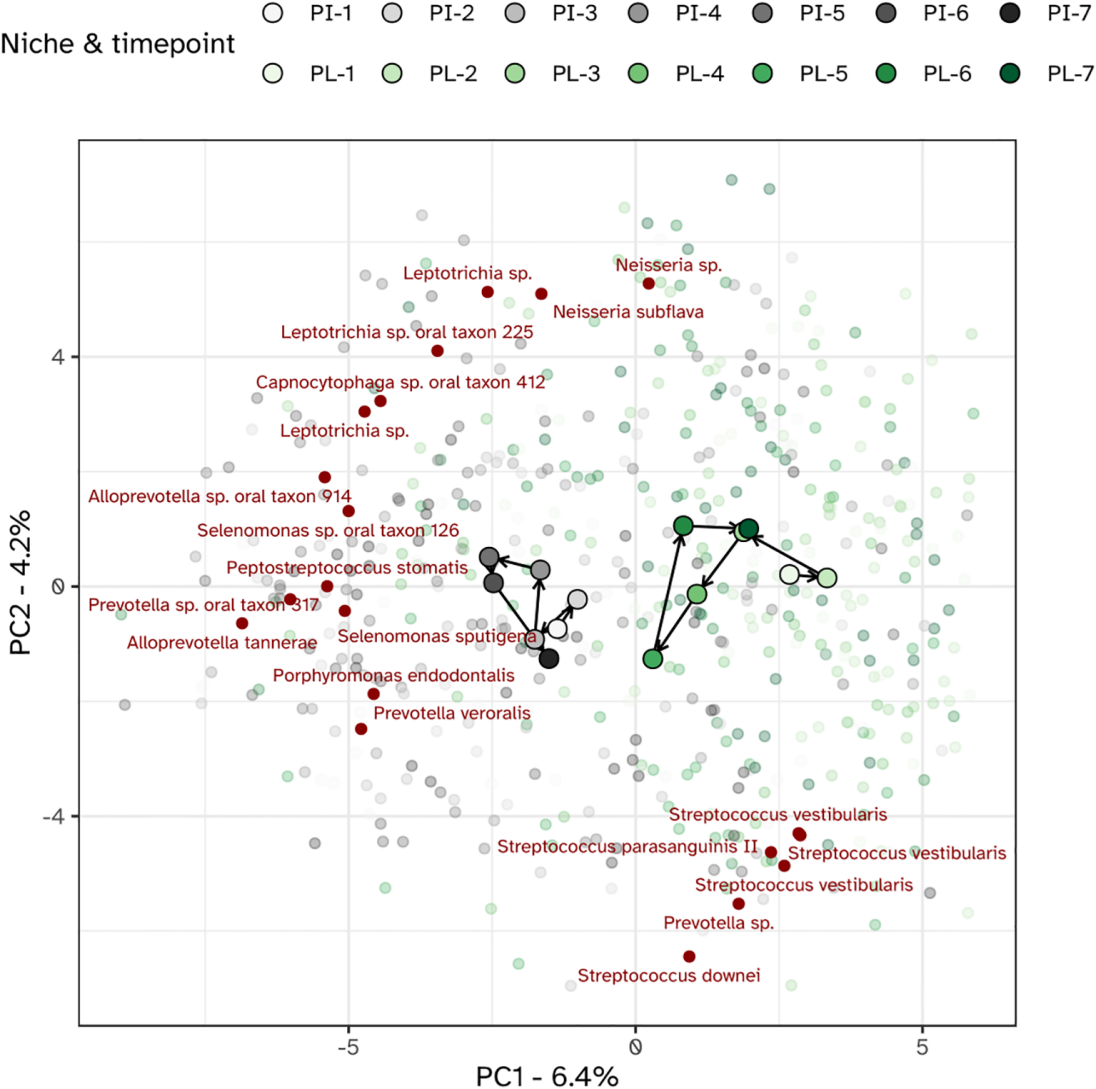
PCA plot of the microbiota of the interproximal plaque (PI) and supragingival plaque (PL). The top 20 MED nodes with the overall strongest association to the ordination axes are shown in red.

Different niches responded differently towards the experimental gingivitis intervention. While the tongue and salivary microbiota displayed little variation between timepoints, larger shifts were observed for the dental plaque and gingival biofilms. During the first 9 days of the intervention, supragingival plaque microbiota directionally moved towards, and became more similar to subgingival and interproximal plaque. Ordination plots for supragingival plaque and interproximal plaque separate from the other niches, indeed revealed the initial shift of the supragingival plaque microbiota along the primary ordination axis, towards interproximal plaque but revealed no further decrease in the beta distance after day 2 (**Figure 7**). Instead, the ordination plot suggested a shift in a different direction between day 2 and day 9, predominantly along the secondary PCA axis and only partially along the primary axis. After day 9, the changes in supragingival plaque microbiota composition renders it to become more similar to the day 2 status. Compositionally, interproximal and supragingival plaque remained different at all time points. Lowest inter-niche beta distance between PL and PI was observed at day 5 (visit 5) (**Supplementary Figure S4**).

### Shifts in bacterial taxa of supragingival plaque during experimental gingivitis

**Figure 8:**
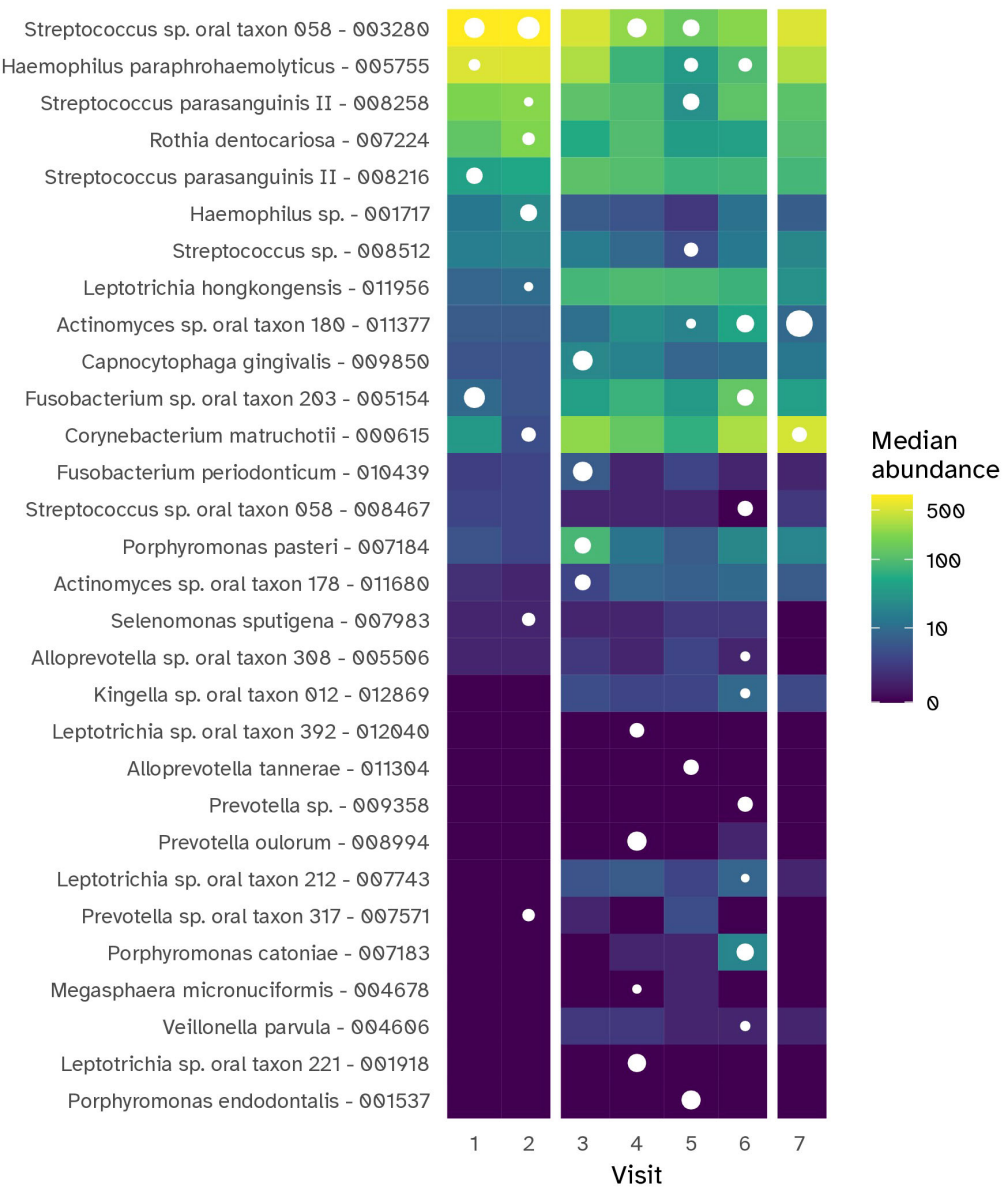
Heat map showing the MEDs (prefixed with taxonomic assignment) that were most representative for each time point of the gingivitis intervention period for the supragingival plaque (PL) niche. The tile color shows the median abundance (number of reads) of a particular MED in this niche. MEDs were selected using Elastic et regression, based on capability of their abundance to help in uniquely discriminating the visit from all other visits. The white dots indicate whether a taxon was selected as discriminative for that timepoint; its size indicates the relative importance in the model.

To investigate the dynamic changes in supragingival plaque composition during experimental gingivitis, a multinomial Elastic-net regression model was employed. This approach enabled the identification of specific taxa that distinguished each time point from the others. In total, 34 MEDs were identified (**Figure 8**; **Supplementary Figure S5**). Several taxa, abundant at baseline, such as *Streptococcus* sp. oral taxon 58, *Haemophilus paraphrohaemolyticus, Rothia dentocariosa* and *Streptococcus parasanguinis* II, declined upon cessation of oral hygiene. For these taxa the lowest relative abundance was noted on day 9. By day 14, the last day of the experimental gingivitis period, their relative abundance had increased and was near or appeared to have returned to baseline levels. Other members of the baseline microbiota increased during the experimental gingivitis period, including *Veillonella dispar, Leptotrichia hongkongensis, Fusobacterium* sp. oral taxon 203, and *Actinomyces* oral taxon 180. A number of MEDs only showed an initial increase in relative abundance at visits 3 (day 2 of experimental gingivitis) or 4 (day 5), and decreasing in relative abundance in subsequent visits. This included *Porphyromonas pasteri*, *Capnocytophaga gingivalis*, and *Fusobacterium periodonticum.* At later stages of the experimental gingivitis period, the supragingival plaque microbiota was characterized by differential abundance of *Alloprevotella sp*. oral taxon 308 at visits 5 and 6 (day 9 and 14 of experimental gingivitis) and *Fusobacterium* sp. oral taxon 203, and *Kingella sp*. oral taxon 012 at visit 6, and by a number of less-abundant bacteria, such as *Leptotrichia* sp., *Prevotella* sp., *Alloprevotella* sp.

### Microbial correlations with immunological and clinical parameters

**Figure 9:**
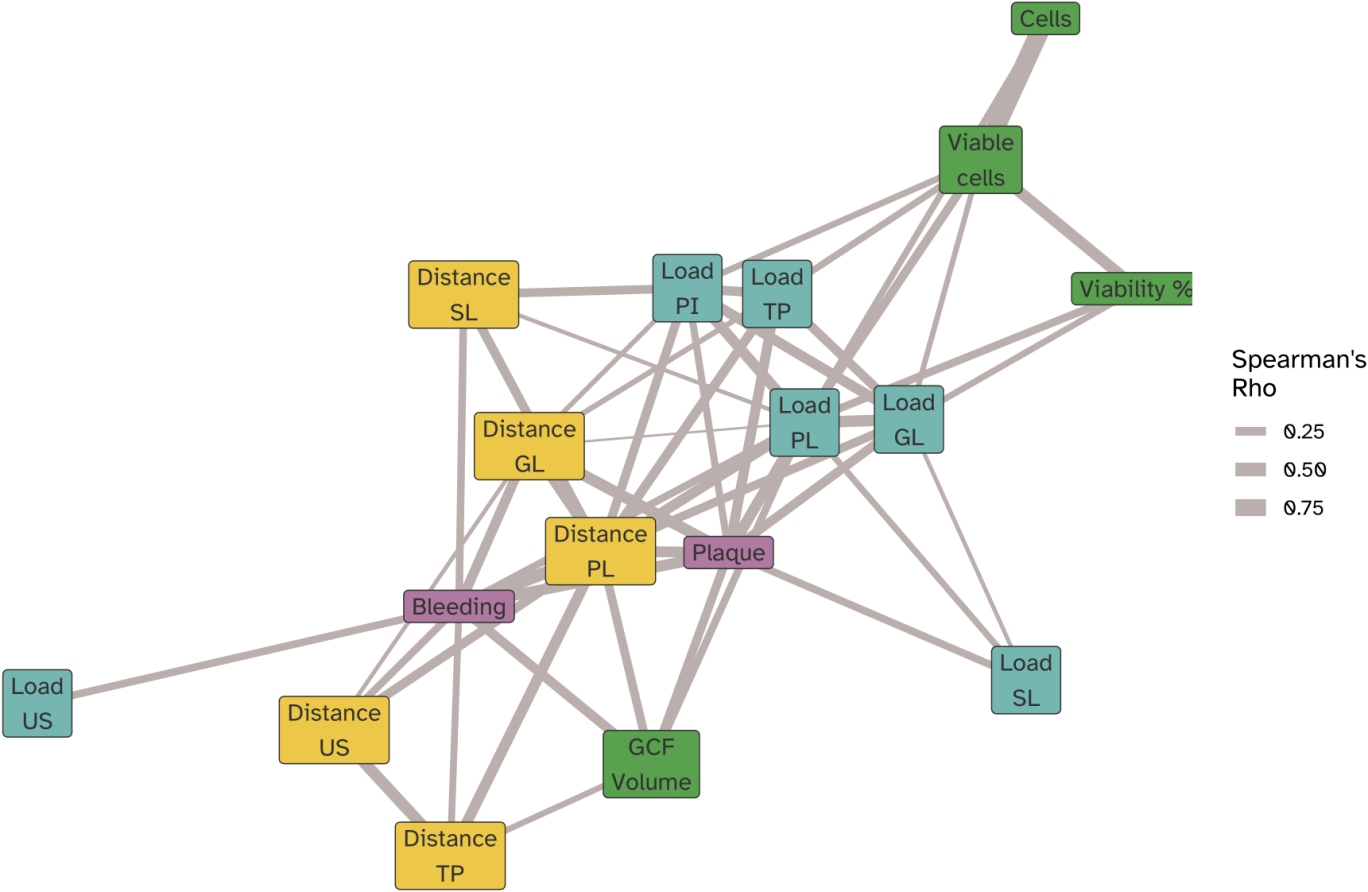
Network correlation (force directed graph) showing the significant (Spearman Rho P<0,05) correlations of oPMN cell counts (cells), viable oPMN counts (Viable cells), and viable fraction of oPMNs (Viability %), BOMP (bleeding) and plaque percentages, GCF volume along with microbiome compositional change as determined by the Aitchison’s distances relative to the baseline (Visit 2) at each visit for the bacterial load in biofilm samples collected for the lower jaw gingiva (distance GL), supragingival plaque (distance PL), subgingival plaque (distance SL), posterior tongue (distance TP) and unstimulated saliva (distance US). Spearman correlations were computed using pairwise complete observations meaning each correlation reflects data from the subset of time points where both variables were present for a given participant. Edges represent the strength of the correlation (|ρ|), and node colors indicate variable categories (clinical, immune, microbial load, microbial distance). Node positions were determined using a force-directed layout, such that strongly correlated variables cluster together.

We next examined the correlations between the bacterial load, changes in microbiota composition and the clinical and immunological parameters hallmarking experimental gingivitis (**Figure 9**; **Supplementary Figure S6**). Plaque percentage correlated positively (Spearman Rho P<0.05) with the bacterial load of all niches, except for saliva. The change in composition of supragingival plaque and the gingival biofilms also correlated with plaque percentage. Percentage of gingival bleeding after marginal probing correlated with the change in biofilm composition of all niches sampled, except for interproximal plaque (PI). The strongest correlation was found with changes in the supragingival plaque (PL), and the tongue biofilm (TP) composition. While the GCF volume also correlated with change in composition of supragingival plaque and that of the tongue, it also revealed a correlation with the bacterial load of supragingival plaque. The oPMN cell count mostly correlated with the bacterial load of supragingival plaque, interproximal plaque and the gingival biofilms, but not to microbial compositional changes (**Figure 9**). oPMN cell counts also did not correlate significantly with BOMP, plaque scores or GCF volume.

**Figure 10:**
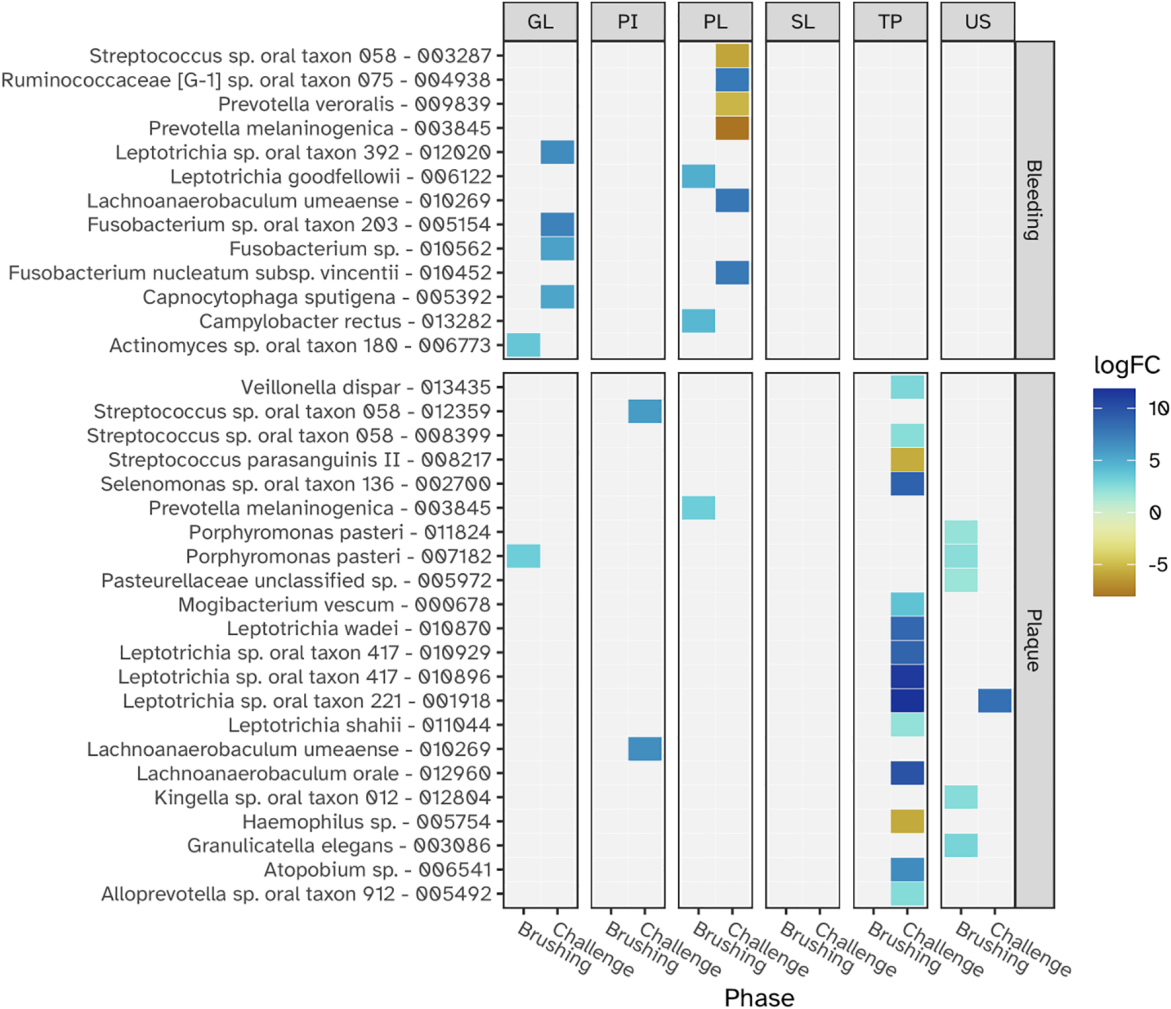
The relationships between individual microbial taxa (MEDs) and the clinical parameters - gingival bleeding and plaque - during the baseline (Brushing phase) and the experimental gingivitis intervention (Challenge phase) at each niche. Panels from left to right show results for the lower jaw gingiva (GL), interproximal plaque (PI), supragingival plaque (PL), subgingival plaque (SL), tongue (TP) and saliva (US) The logFC value shows how taxonomic abundance changed with a one unit increase of bleeding and plaque. Bleeding is expressed as the square-root of the fraction of sites with gingival bleeding of all examined sites, while plaque is expressed as the square of the fraction of sites with plaque of all examined sites. Only logFC values with a Benjamini-Hochberg adjusted p-value < 0.05 are shown.

We next examined differential abundance of individual taxa of the oral microbiome linked to the gingival bleeding and plaque percentages. Data collected during baseline, representing the natural state of gingiva, were analyzed separately from the experimental gingivitis phase. Crucially, we found no overlap in microbial taxa that exhibited significant correlation with gingival bleeding or plaque and also no overlap between the baseline (V1-2) and the experimental gingivitis period (V3-V6) (**Figure 10**). For gingival bleeding, significant correlations were found for microbial taxa of supragingival plaque (PL) and the gingival biofilms (GL), but not for taxa in the other niches. For plaque, most correlations were found for the tongue biofilms, showing an overrepresentation of *Leptotrichia* in positive correlations. Most of the taxa showing correlations with the number or percentage of viable oPMNs were for the taxa found in saliva (US) and supragingival plaque (PL) (**Supplementary Table 1**). No individual taxa correlated significantly to GCF volume at either baseline or during the intervention.

## Discussion

This study aimed to gain a deeper understanding of the community-level associations of the oral microbiome in the transition from what are considered homeostatic to dysbiotic microbial communities. We utilized the well-established experimental gingivitis model to investigate the microbial ecological dynamics within various intra-oral niches in a cohort of healthy volunteers, examining their correlation with clinical and immunological factors (Loe et al., 1965). This model offered a standardized approach to track the buildup and maturation of each participant’s microbiota alongside gingival inflammation, enabling temporal analysis of microbial changes and clinical outcomes like plaque and bleeding. While two weeks without oral hygiene does not reflect typical real-world conditions, it allowed us to examine intra-individual microbial and inflammatory responses under reproducible experimental settings. Several recent studies used a similar approach, enabling the identification of immunological and microbial response patterns, possibly underlying inter-individual variation in susceptibility towards gingival inflammation (Nowicki et al., 2018; Nascimento et al., 2019; Bamashmous et al., 2021; Huang et al., 2021; Kerns et al., 2023). Studies thus far were focused on supragingival and subgingival plaque biofilms; few studies also included other oral niches (Hall et al., 2023).

In line with previous observations, the experimental gingivitis intervention resulted in significant increases in plaque and gingival bleeding. These changes were accompanied by alterations in the volume of gingival crevicular fluid (GCF) and the number and vitality of oral polymorphonuclear neutrophils (oPMNs). The observed increase in GCF volume on day 14 hallmarks the inflammatory response associated with gingivitis. Conversely, the transient elevation in oPMNs at day 9 and the subsequent decline at day 14 has not been observed before and could reflect a dynamic phase in the release, or activation of immune cells during the earlier stages of experimental gingivitis. This dynamic behavior was also reflected in the microbial biofilm dynamics.

The investigation of microbial ecological dynamics in response to experimental gingivitis revealed intricate changes across different oral niches. Bacterial load analysis demonstrated increased level of microbial biomass in supragingival plaque at all timepoints during experimental gingivitis. In the resolution phase, these levels declined again, but remained higher than baseline levels. Subgingival biomass was significantly higher at day 14. Importantly, the biomass of interproximal plaque, and gingival biofilms was elevated only in the early phases of experimental gingivitis but not at day 14, while bacterial biomass of tongue and salivary samples did not change significantly. Microbial diversity analyses provided further insights into the ecological dynamics. An increase in alpha diversity was observed during experimental gingivitis in multiple niches. While the subgingival biofilms only showed an increase in diversity towards day 14 of the experimental gingivitis intervention, other niches, such as the tongue, interproximal plaque and the gingival biofilm, displayed a transient increase in the early phase of the intervention. Beta diversity analyses indicated niche-specific and non-linear shifts in microbial composition during the induction phase of gingivitis. Supragingival plaque displayed an increase in beta diversity during the first five days and remained elevated through the remainder of the study. For several niches, including subgingival plaque, the gingival biofilm, interproximal plaque and posterior tongue, the beta diversity increased during the initial phases of experimental gingivitis, but decreased again in later stages. The transient and dynamic ecological behavior is further exemplified by the changes of supragingival plaque composition. During induction of experimental gingivitis, the supragingival plaque composition became more similar to that of interproximal plaque, but remained distinct in composition. Changes included a decline in a number of abundant commensal species, including *Streptococcus*, coinciding with an increase in the relative abundance of *Leptotrichia, Prevotella* and *Fusobacterium*. These findings are in line with previous studies, mapping taxonomic shifts during experimental gingivitis and where it enabled defining high and low clinical response groups (Bamashmous et al., 2021; Hall et al., 2023; Kerns et al., 2023). A feature previously not highlighted is the high degree of nonlinear behavior on the ecosystem. A preceding analysis of the salivary metabolome of samples retrieved from this study also revealed an early and transient increase in the abundance of several metabolites (Prodan et al., 2016). This included products formed by microbial amino acid fermentation, such as cadaverine and α-hydroxyisovalerate, mevalonate, as well as transient changes in several lipid concentrations, including lactosylceramide (lactosyl-N-palmitoyl-sphingosine). The observed non-linear shifts in microbial composition, diversity, and abundance during experimental gingivitis raise the question of occurrence of potential ecological tipping points within the oral microbiota. The dynamics of taxa abundances, including increases in some taxa and decreases in others, suggest a complex interplay that could resemble shifts associated with ecological tipping points. An ecological tipping point in a microbial ecosystem represents a critical threshold where complex factors such as disrupted community structure, environmental changes, resource limitations, feedback loops, and disturbances lead to rapid and potentially irreversible shifts in microbial composition and function (Scheffer et al., 2009). However, we were unable to identify taxa showing bistable abundance distributions which is believed to be a key attribute of ecological tipping point behavior (Lahti et al., 2014).

### Correlations between microbial ecological dynamics and clinical and immunological parameters

Examining relationships between the microbial ecological changes during experimental gingivitis and the clinical and immunological parameters suggested that microbial load and compositional change attribute independently to plaque, gingival bleeding, GCF volume and increase in oPMN cell numbers. Presence of plaque is mostly related to bacterial biofilm density (bacterial load) of supragingival and interproximal biofilms, but also that of other niches including the tongue. The lack of correlation between plaque scores and salivary bacterial load may reflect that saliva is not a microbial niche itself, but a suspension of microbes primarily shed from soft tissue surfaces — particularly the tongue — as also reported in previous studies (Mark Welch et al., 2019; Esberg et al., 2022), and confirmed by our ordination analysis (**Figure 6**). Gingival bleeding (BOMP%) also showed a significant correlation with the bacterial load of supragingival plaque, but was primarily associated to compositional changes of the various niches, including that of supragingival and interproximal plaque and the tongue. The increase in GCF volume showed a positive correlation to both plaque and bleeding scores as well as the bacterial load and compositional change of supragingival plaque. This may suggest that despite the well-established correlation between plaque and gingival bleeding, these may to some extent, at least from a microbial perspective, be independent parameters and may relate differently to biofilm bacterial density and biofilm composition (Breuer and Cosgrove, 1989), a finding that aligns with earlier studies showing inter-individual variation in the inflammatory response to plaque (Abbas et al., 1986; Galgut, 1988; Badersten et al., 1990). The uncovered taxonomic relations with plaque related to taxa of the tongue biofilm, mostly belonging to the genus *Leptotrichia*. Although *Leptotrichia* has been associated with gingivitis (Eribe and Olsen, 2017) and has been demonstrated to play a role in biofilm structure (Mark Welch et al., 2016), we have no explanation on how specific taxa on the tongue could influence plaque presence of teeth. Gingival bleeding related to specific taxa of supragingival plaque. In line with previous observations, a negative association was found for *Streptococcus* (Bloch et al., 2024).

Strengths of the study are that the study employed a comprehensive design by investigating clinical, immunological, and microbiological aspects of experimental gingivitis. This multidimensional approach provides a more holistic understanding of the condition. Participants were followed over multiple time points, allowing for the assessment of changes and trends during the induction and resolution phases of experimental gingivitis. This longitudinal design increases the validity of the findings. The study analyzed microbial samples from various oral niches, including supragingival and subgingival plaque, interproximal plaque, saliva, tongue, and gingiva. This diversity provides a broader perspective on the impact of gingivitis on the oral ecosystem. Weaknesses of the study are the relatively small number of participants, which could limit the generalizability of the findings to larger populations. The two-week experimental gingivitis period might not fully capture the long-term effects of gingivitis on clinical, immunological, and microbial parameters. As oral biofilm sampling for microbiota analysis disturbed plaque development, different dental and gingival sites were used to sample at the different timepoints. Although adjacent and anatomically similar oral niches were chosen, it may foresee an additional source for variation in our dataset. While 16S rRNA gene sequencing provides valuable insights into microbial composition, it has limitations in accurately identifying certain species and strains. Metagenomic analysis or shotgun sequencing could provide a more detailed understanding of microbial changes. While the study demonstrated associations between clinical, immunological, and microbial parameters, it cannot establish a causal relationship between these factors and experimental gingivitis. There might be selection bias in the recruitment of participants, as healthy volunteers might not fully represent the oral health status of the general population. The study reinitiated oral hygiene and followed the study participants for only one week after the experimental gingivitis period, resulting in incomplete recovery. While clinical plaque scores returned to near-baseline levels, bleeding scores, bacterial load, alpha and beta diversity remained elevated. This apparent divergence likely reflects differences in how these parameters are measured: plaque scores assess the presence of visible plaque across multiple sites, whereas bacterial load quantifies microbial genome content at a specific sampled site. Residual biomass may persist despite visual removal, underscoring the value of integrating clinical and molecular assessments A one-week resolution phase was chosen to capture early clinical and microbial recovery, based on prior studies showing that plaque and bleeding indices typically begin to reverse within this timeframe (Van der Weijden et al., 1994; Hall et al., 2023). This design also allowed us to explore potential delays in microbiome normalization. A longer resolution phase might provide more insights into the recovery of oral health parameters.

It is important to note that our findings reflect associations between microbial composition and clinical parameters rather than causative relationships. While temporal correlations suggest coordinated changes during gingival inflammation, the observational nature of this study does not permit causal inference.

## Conclusions

This study highlights the intricate and dynamic nature of microbial changes during gingival inflammation, demonstrating that plaque and gingival bleeding are independent clinical parameters with distinct microbial underpinnings. Plaque was strongly associated with bacterial load, particularly in supragingival and interproximal niches, while gingival bleeding correlated more with compositional shifts in supragingival plaque and the tongue microbiota. These findings emphasize the importance of relationships between multiple niches in shaping clinical outcomes. The tongue emerged as a significant contributor to gingival bleeding, underscoring the need to consider its microbiota in future studies and clinical interventions. Furthermore, the observed nonlinear microbial dynamics, including abrupt shifts in community composition, suggest a complex ecological behavior that may approach tipping points. While the persistence of some microbial changes after hygiene restoration indicates potential lasting effects of dysbiosis, the niche-specific insights gained here provide a clearer understanding of microbial diversity and taxonomic shifts. Together, these findings advance the understanding of gingival dysbiosis and pave the way for developing targeted interventions to restore microbial balance and manage periodontal health effectively.

## Data Availability

The datasets presented in this study can be found in online repositories. The names of the repository/repositories and accession number(s) can be found below: https://www.ebi.ac.uk/ena, PRJEB83572.

## References

[B1] Abbas F. Van der Velden U. Hart A. A. Moorer W. R. Vroom T. M. Scholte G. (1986). Bleeding/plaque ratio and the development of gingival inflammation. J. Clin. Periodontol 13, 774–782. doi: 10.1111/j.1600-051X.1986.tb00881.x 3490497

[B2] al-Essa L. Niwa M. Kohno K. Tsurumi K. (1994). A proposal for purification of salivary polymorphonuclear leukocytes by combination of nylon mesh filtration and density-gradient method: a validation by superoxide- and cyclic AMP-generating responses. Life Sci. 55, Pl333–Pl338. doi: 10.1016/0024-3205(94)00773-X 7934636

[B3] Arimatsu K. Yamada H. Miyazawa H. Minagawa T. Nakajima M. Ryder M. I.. (2014). Oral pathobiont induces systemic inflammation and metabolic changes associated with alteration of gut microbiota. Sci. Rep. 4, 4828. doi: 10.1038/srep04828 24797416 PMC4010932

[B4] Ashkenazi M. Dennison D. K. (1989). A new method for isolation of salivary neutrophils and determination of their functional activity. J. Dent. Res. 68, 1256–1261. doi: 10.1177/00220345890680080901 2576658

[B5] Badersten A. Nilvéus R. Egelberg J. (1990). Scores of plaque, bleeding, suppuration and probing depth to predict probing attachment loss. 5 years of observation following nonsurgical periodontal therapy. J. Clin. Periodontol 17, 102–107. doi: 10.1111/j.1600-051X.1990.tb01070.x 2406291

[B6] Bamashmous S. Kotsakis G. A. Kerns K. A. Leroux B. G. Zenobia C. Chen D.. (2021). Human variation in gingival inflammation. Proc. Natl. Acad. Sci. U.S.A. 118, e2012578118. doi: 10.1073/pnas.2012578118 34193520 PMC8271746

[B7] Bates D. Mächler M. Bolker B. Walker S. (2015). Fitting linear mixed-effects models using lme4. J. Stat. Software 67, 1–48. doi: 10.18637/jss.v067.i01

[B8] Benjamini Y. Hochberg Y. (1995). Controlling the false discovery rate: A practical and powerful approach to multiple testing. J. R. Stat. Society Ser. B (Methodological) 57, 289–300. doi: 10.1111/j.2517-6161.1995.tb02031.x

[B9] Bloch S. Hager-Mair F. F. Andrukhov O. Schaffer C. (2024). Oral streptococci: modulators of health and disease. Front. Cell Infect. Microbiol. 14, 1357631. doi: 10.3389/fcimb.2024.1357631 38456080 PMC10917908

[B10] Breuer M. M. Cosgrove R. S. (1989). The relationship between gingivitis and plaque levels. J. Periodontol 60, 172–175. doi: 10.1902/jop.1989.60.4.172 2724030

[B11] Chen T. Yu W. H. Izard J. Baranova O. V. Lakshmanan A. Dewhirst F. E. (2010). The Human Oral Microbiome Database: a web accessible resource for investigating oral microbe taxonomic and genomic information. Database (Oxford) 2010, baq013. doi: 10.1093/database/baq013 20624719 PMC2911848

[B12] Dewhirst F. E. Chen T. Izard J. Paster B. J. Tanner A. C. Yu W. H.. (2010). The human oral microbiome. J. Bacteriol 192, 5002–5017. doi: 10.1128/JB.00542-10 20656903 PMC2944498

[B13] Edgar R. C. Haas B. J. Clemente J. C. Quince C. Knight R. (2011). UCHIME improves sensitivity and speed of chimera detection. Bioinformatics 27, 2194–2200. doi: 10.1093/bioinformatics/btr381 21700674 PMC3150044

[B14] Eren A. M. Morrison H. G. Lescault P. J. Reveillaud J. Vineis J. H. Sogin M. L.. (2015). Minimum entropy decomposition: unsupervised oligotyping for sensitive partitioning of high-throughput marker gene sequences. Isme J. 9, 968–979. doi: 10.1038/ismej.2014.195 25325381 PMC4817710

[B15] Eribe E. R. K. Olsen I. (2017). Leptotrichia species in human infections II. J. Oral. Microbiol. 9, 1368848. doi: 10.1080/20002297.2017.1368848 29081911 PMC5646626

[B16] Esberg A. Eriksson L. Johansson I. (2022). Site- and time-dependent compositional shifts in oral microbiota communities. Front. Oral. Health 3, 826996. doi: 10.3389/froh.2022.826996 35300180 PMC8921071

[B17] Fluitman K. S. van den Broek T. J. Nieuwdorp M. Visser M. IJzerman R. G. Keijser B. J. F.. (2021). Associations of the oral microbiota and Candida with taste, smell, appetite and undernutrition in older adults. Sci. Rep. 11, 23254. doi: 10.1038/s41598-021-02558-8 34853371 PMC8636608

[B18] Friedman J. Hastie T. Tibshirani R. (2010). Regularization paths for generalized linear models via coordinate descent. J. Stat. Softw 33, 1–22. doi: 10.18637/jss.v033.i01 20808728 PMC2929880

[B19] Galgut P. N. (1988). The bleeding/plaque ratio in the treatment of periodontal disease. J. Clin. Periodontol 15, 606–611. doi: 10.1111/j.1600-051X.1988.tb02259.x 3264293

[B20] Greenacre M. (2021). Compositional data analysis. Annu. Rev. Stat Its Appl. 8, 271–299. doi: 10.1146/annurev-statistics-042720-124436

[B21] Hall M. W. Wellappuli N. C. Huang R. C. Wu K. Lam D. K. Glogauer M.. (2023). Suspension of oral hygiene practices highlights key bacterial shifts in saliva, tongue, and tooth plaque during gingival inflammation and resolution. ISME Commun. 3, 23. doi: 10.1038/s43705-023-00229-5 36966246 PMC10039884

[B22] Hoffman G. E. Roussos P. (2021). Dream: powerful differential expression analysis for repeated measures designs. Bioinformatics. 37 (2), 192–201. doi: 10.1093/bioinformatics/btaa687 32730587 PMC8055218

[B23] Hoffman G. E. Schadt E. E. (2016). variancePartition: interpreting drivers of variation in complex gene expression studies. BMC Bioinformatics 17, 483. doi: 10.1186/s12859-016-1323-z 27884101 PMC5123296

[B24] Huang S. He T. Yue F. Xu X. Wang L. Zhu P.. (2021). Longitudinal multi-omics and microbiome meta-analysis identify an asymptomatic gingival state that links gingivitis, periodontitis, and aging. mBio 12, e03281-20. doi: 10.1128/mBio.03281-20 33688007 PMC8092283

[B25] ISO (2016). Dentistry -Designation system for teeth and areas of the oral cavity (Geneva, Switzerland: International Organization for Standardization), 4.

[B26] Kassambara A. (2023). Rstatix: Pipe-Friendly Framework for Basic Statistical Tests. R package version 0.7.2. Available online at: https://rpkgs.datanovia.com/rstatix/.

[B27] Kassebaum N. J. Smith A. G. C. Bernabé E. Fleming T. D. Reynolds A. E. Vos T.. (2017). Global, regional, and national prevalence, incidence, and disability-adjusted life years for oral conditions for 195 countries, 1990-2015: A systematic analysis for the global burden of diseases, injuries, and risk factors. J. Dent. Res. 96, 380–387. doi: 10.1177/0022034517693566 28792274 PMC5912207

[B28] Kerns K. A. Bamashmous S. Hendrickson E. L. Kotsakis G. A. Leroux B. G. Daubert D. D.. (2023). Localized microbially induced inflammation influences distant healthy tissues in the human oral cavity. Proc. Natl. Acad. Sci. U.S.A. 120, e2306020120. doi: 10.1073/pnas.2306020120 37782795 PMC10576129

[B29] Kilian M. Chapple I. L. Hannig M. Marsh P. D. Meuric V. Pedersen A. M.. (2016). The oral microbiome - an update for oral healthcare professionals. Br. Dent. J. 221, 657–666. doi: 10.1038/sj.bdj.2016.865 27857087

[B30] Kistler J. O. Booth V. Bradshaw D. J. Wade W. G. (2013). Bacterial community development in experimental gingivitis. PLoS One 8, e71227. doi: 10.1371/journal.pone.0071227 23967169 PMC3743832

[B31] Kong Y. (2011). Btrim: A fast, lightweight adapter and quality trimming program for next-generation sequencing technologies. Genomics 98, 152–153. doi: 10.1016/j.ygeno.2011.05.009 21651976

[B32] Kramer C. D. Genco C. A. (2017). Microbiota, immune subversion, and chronic inflammation. Front. Immunol. 8, 255. doi: 10.3389/fimmu.2017.00255 28348558 PMC5346547

[B33] Kuhn M. (2008). Building predictive models in R using the caret package. J. Stat. Software 28, 1–26. doi: 10.18637/jss.v028.i05

[B34] Kumar P. S. (2017). From focal sepsis to periodontal medicine: a century of exploring the role of the oral microbiome in systemic disease. J. Physiol. 595, 465–476. doi: 10.1113/tjp.2017.595.issue-2 27426277 PMC5233655

[B35] Lahti L. Salojärvi J. Salonen A. Scheffer M. de Vos W. M. (2014). Tipping elements in the human intestinal ecosystem. Nat. Commun. 5, 4344. doi: 10.1038/ncomms5344 25003530 PMC4102116

[B36] Lamont R. J. Koo H. Hajishengallis G. (2018). The oral microbiota: dynamic communities and host interactions. Nat. Rev. Microbiol. 16, 745–759. doi: 10.1038/s41579-018-0089-x 30301974 PMC6278837

[B37] Lenth R. V. (2022). emmeans: Estimated Marginal Means, aka Least-Squares Means. R package version 1.11.1-00001, Available online at: https://rvlenth.github.io/emmeans/.

[B38] Loe H. Theilade E. Jensen S. B. (1965). Experimental gingivitis in man. J. Periodontol (1930) 36, 177–187. doi: 10.1902/jper.1965.36.issue-3 14296927

[B39] Mark Welch J. L. Dewhirst F. E. Borisy G. G. (2019). Biogeography of the oral microbiome: the site-specialist hypothesis. Annu. Rev. Microbiol. 73, 335–358. doi: 10.1146/annurev-micro-090817-062503 31180804 PMC7153577

[B40] Mark Welch J. L. Rossetti B. J. Rieken C. W. Dewhirst F. E. Borisy G. G. (2016). Biogeography of a human oral microbiome at the micron scale. Proc. Natl. Acad. Sci. U.S.A. 113, E791–E800. doi: 10.1073/pnas.1522149113 26811460 PMC4760785

[B41] Nascimento G. G. Danielsen B. Baelum V. Lopez R. (2019). Identification of inflammatory response patterns in experimental gingivitis studies. Eur. J. Oral. Sci. 127, 33–39. doi: 10.1111/eos.2019.127.issue-1 30412312

[B42] Nowicki E. M. Shroff R. Singleton J. A. Renaud D. E. Wallace D. Drury J.. (2018). Microbiota and metatranscriptome changes accompanying the onset of gingivitis. mBio 9, e00575-18. doi: 10.1128/mBio.00575-18 29666288 PMC5904416

[B43] Prodan A. Imangaliyev S. Brand H. S. Rosema N. A. M. Leving E. Crielaard W. C.. (2016). Effect of experimental gingivitis induction and erythritol on the salivary metabolome and functional biochemistry of systemically healthy young adults. Metabolomics 12, 147. doi: 10.1007/s11306-016-1096-4

[B44] R core team (2022). R: A language and environment for statistical computing (Vienna, Austria: R Foundation for Statistical Computing).

[B45] Rijkschroeff P. Jansen I. D. van der Weijden F. A. Keijser B. J. Loos B. G. Nicu E. A.. (2016). Oral polymorphonuclear neutrophil characteristics in relation to oral health: a cross-sectional, observational clinical study. Int. J. Oral. Sci. 8, 191–198. doi: 10.1038/ijos.2016.23 27515277 PMC5113092

[B46] Rijkschroeff P. Schoenmaker T. Caspers M. Verschuren L. Keijser B. J. F. Nicu E. A.. (2020). Dentistry and OMICS: transcriptome dynamics of an oral ecosystem as measured by changes in oral polymorphonuclear neutrophils in experimental gingivitis. Omics 24, 531–540. doi: 10.1089/omi.2020.0034 32559408

[B47] Scheffer M. Bascompte J. Brock W. A. Brovkin V. Carpenter S. R. Dakos V.. (2009). Early-warning signals for critical transitions. Nature 461, 53–59. doi: 10.1038/nature08227 19727193

[B48] Schloss P. D. Westcott S. L. Ryabin T. Hall J. R. Hartmann M. Hollister E. B.. (2009). Introducing mothur: open-source, platform-independent, community-supported software for describing and comparing microbial communities. Appl. Environ. Microbiol. 75, 7537–7541. doi: 10.1128/AEM.01541-09 19801464 PMC2786419

[B49] Silness J. Loe H. (1964). Periodontal disease in pregnancy. Ii. Correlation between oral hygiene and periodontal condtion. Acta Odontol Scand. 22, 121–135. doi: 10.3109/00016356408993968 14158464

[B50] Sultan A. S. Kong E. F. Rizk A. M. Jabra-Rizk M. A. (2018). The oral microbiome: A Lesson in coexistence. PloS Pathog. 14, e1006719. doi: 10.1371/journal.ppat.1006719 29370304 PMC5784999

[B51] Takeshita T. Kageyama S. Furuta M. Tsuboi H. Takeuchi K. Shibata Y.. (2016). Bacterial diversity in saliva and oral health-related conditions: the Hisayama Study. Sci. Rep. 6, 22164. doi: 10.1038/srep22164 26907866 PMC4764907

[B52] The Human Microbiome Project Consortium (2012). Structure, function and diversity of the healthy human microbiome. Nature 486, 207–214. doi: 10.1038/nature11234 22699609 PMC3564958

[B53] van der Veen M. H. Volgenant C. M. Keijser B. Ten Cate J. B. Crielaard W. (2016). Dynamics of red fluorescent dental plaque during experimental gingivitis–A cohort study. J. Dent. 48, 71–76. doi: 10.1016/j.jdent.2016.02.010 26921667

[B54] Van der Weijden G. A. Timmerman M. F. Nijboer A. Reijerse E. Van der Velden U. (1994). Comparison of different approaches to assess bleeding on probing as indicators of gingivitis. J. Clin. Periodontol 21, 589–594. doi: 10.1111/j.1600-051X.1994.tb00748.x 7806674

[B55] Wade W. G. (2013). The oral microbiome in health and disease. Pharmacol. Res. 69, 137–143. doi: 10.1016/j.phrs.2012.11.006 23201354

[B56] Wickham H. (2016). ggplot2: Elegant Graphics for Data Analysis (New York: Springer-Verlag). doi: 10.1007/978-3-319-24277-4_9

[B57] Zaura E. Brandt B. W. Prodan A. Teixeira de Mattos M. J. Imangaliyev S. Kool J.. (2017). On the ecosystemic network of saliva in healthy young adults. ISME J. 11, 1218–1231. doi: 10.1038/ismej.2016.199 28072421 PMC5475835

