# Supplementary material for: Oral bacterial community dynamics during induction of gingival inflammation
Source: Front Cell Infect Microbiol. 2025 Jun 16;15:1597690. doi: 10.3389/fcimb.2025.1597690 (PMC12206739; doi:10.3389/fcimb.2025.1597690)
Supplement: Supplementary file 1 [file Supplementaryfile1.docx]

# Supplementary Material


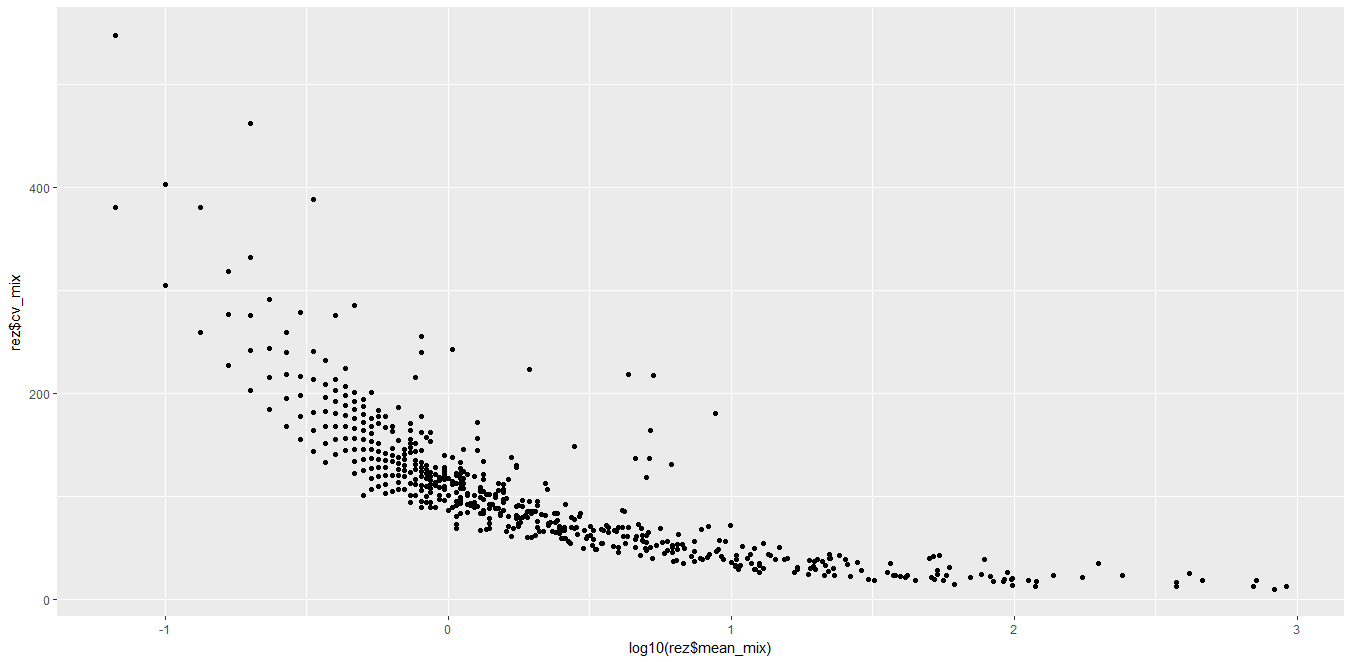


**Supplementary Figure 1**. Coefficient of Variation (CV) for Mean Reads per Sample (Log10 Scale)

The coefficient of variation (CV) for mean reads per sample across the 30 sequencing runs for all (MEDs) in the Quality Control (QC) sample mix. The x-axis represents the mean reads per sample on a log10 scale, while the y-axis shows the CV values for each MED. Each point represents an individual MED, with its position reflecting the mean read abundance and variability across sequencing runs. The log10 scale enables visualization of the full range of MED abundances, including those with low read counts.


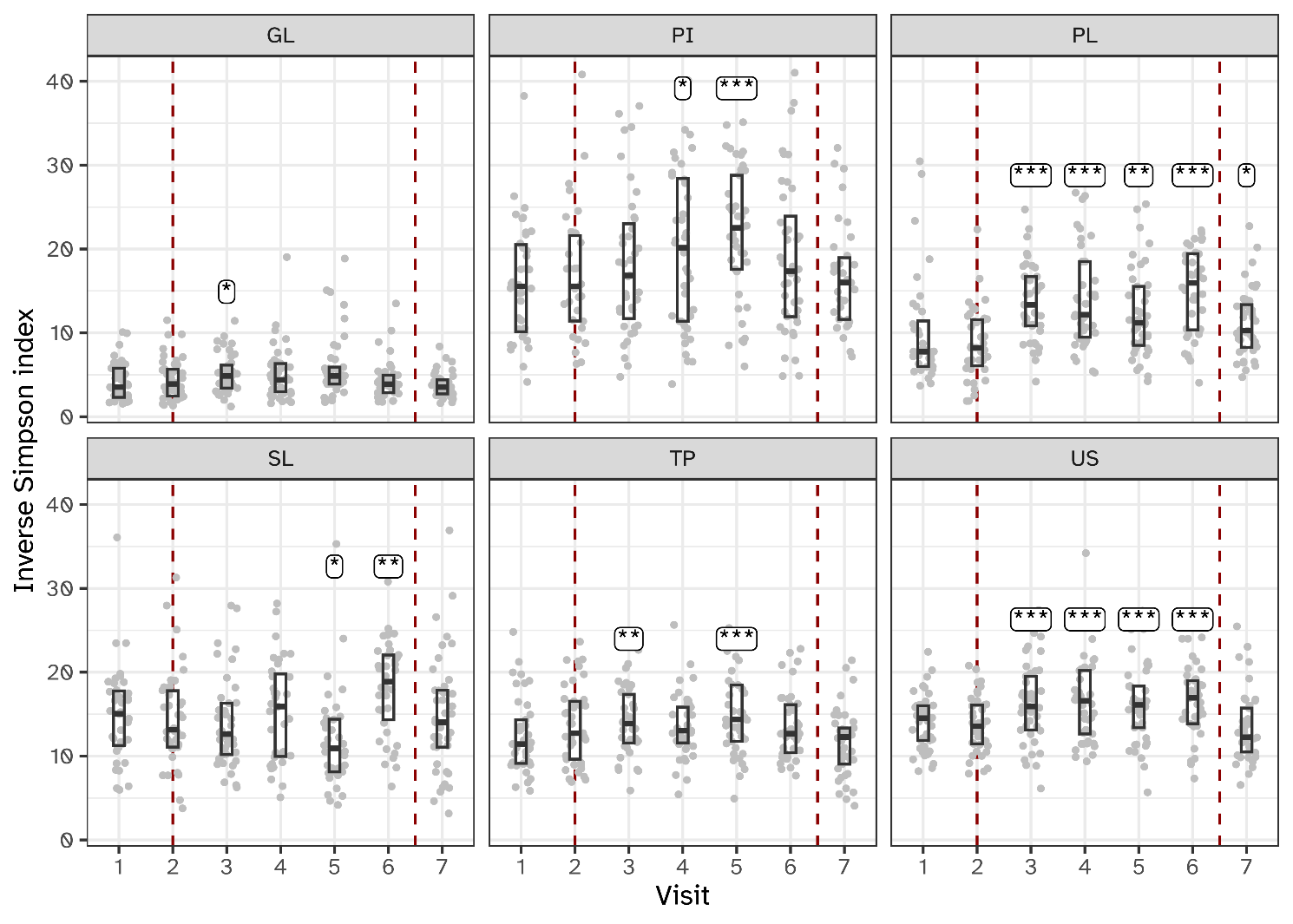
**Supplementary Figure 2**. Boxplots showing microbial alpha diversity, as determined by the inverse Simpson index of the six niches. Annotations indicate a significant difference versus visit 2 (***: P < 0.001; **: P < 0.01; *: P < 0.05). The microbiota of six oral niches sampled were: lower jaw gingiva (GL), interproximal plaque (PI), supragingival plaque (PL), subgingival plaque (SL), posterior tongue (TP) and unstimulated saliva (US). Visits 1 and 2 - baseline, visit 3 - day 2, visit 4 - day 5, visit 5 - day 9 and visit 6 - day 14 of experimental gingivitis, visit 7 - day 7 of the resolution phase.


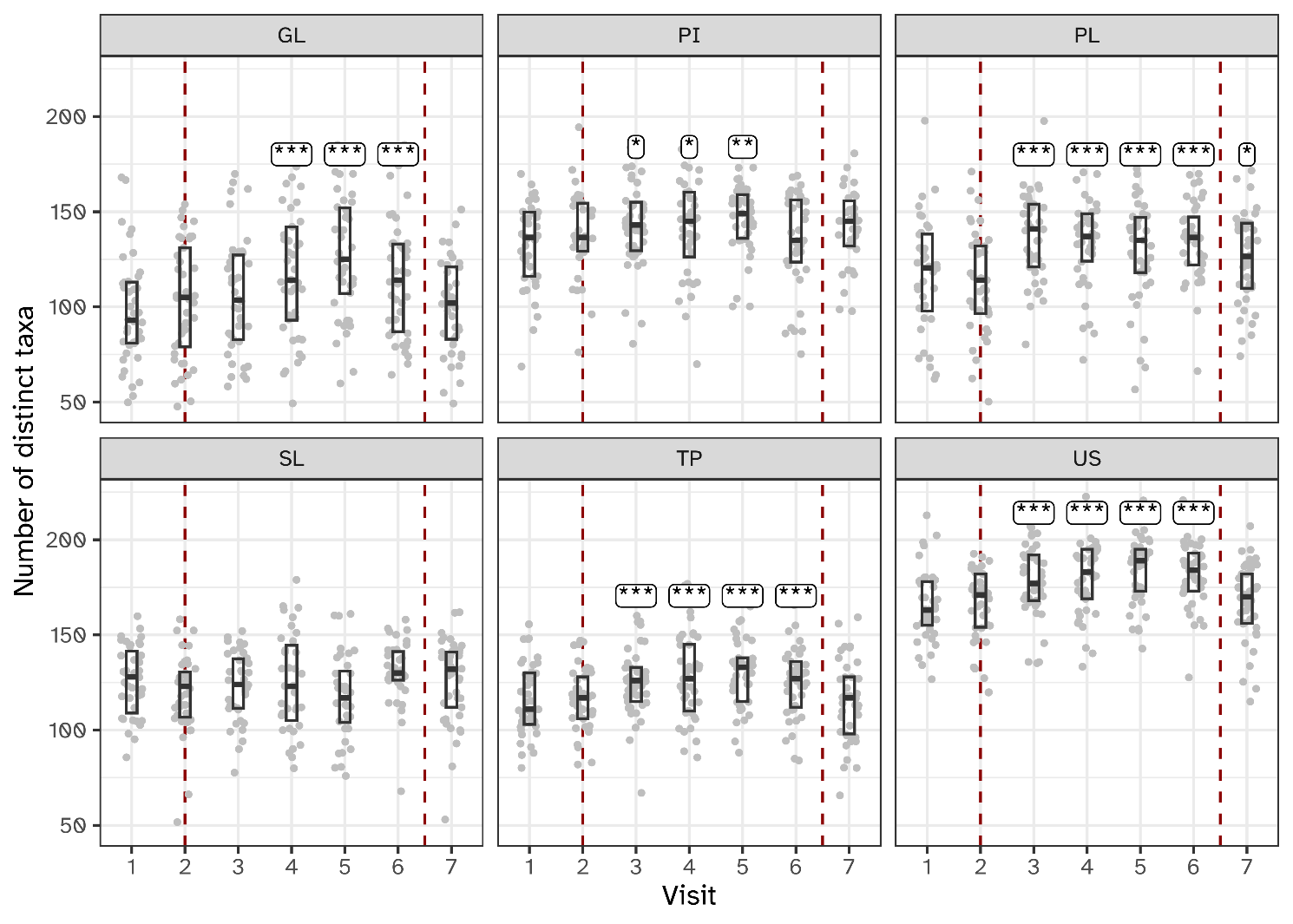
**Supplementary Figure 3.** Boxplots showing the normalized counts of the number of unique MEDs with a count > 0 found at each timepoint for each of the six niches. Annotations indicate a significant difference versus visit 2 (***: P < 0.001; **: P < 0.01; *: P < 0.05). The microbiota of six oral niches sampled were: lower jaw gingiva (GL), interproximal plaque (PI), supragingival plaque (PL), subgingival plaque (SL), posterior tongue (TP) and unstimulated saliva (US). Visits 1 and 2 - baseline, visit 3 - day 2, visit 4 - day 5, visit 5 - day 9 and visit 6 - day 14 of experimental gingivitis, visit 7 - day 7 of the resolution phase.


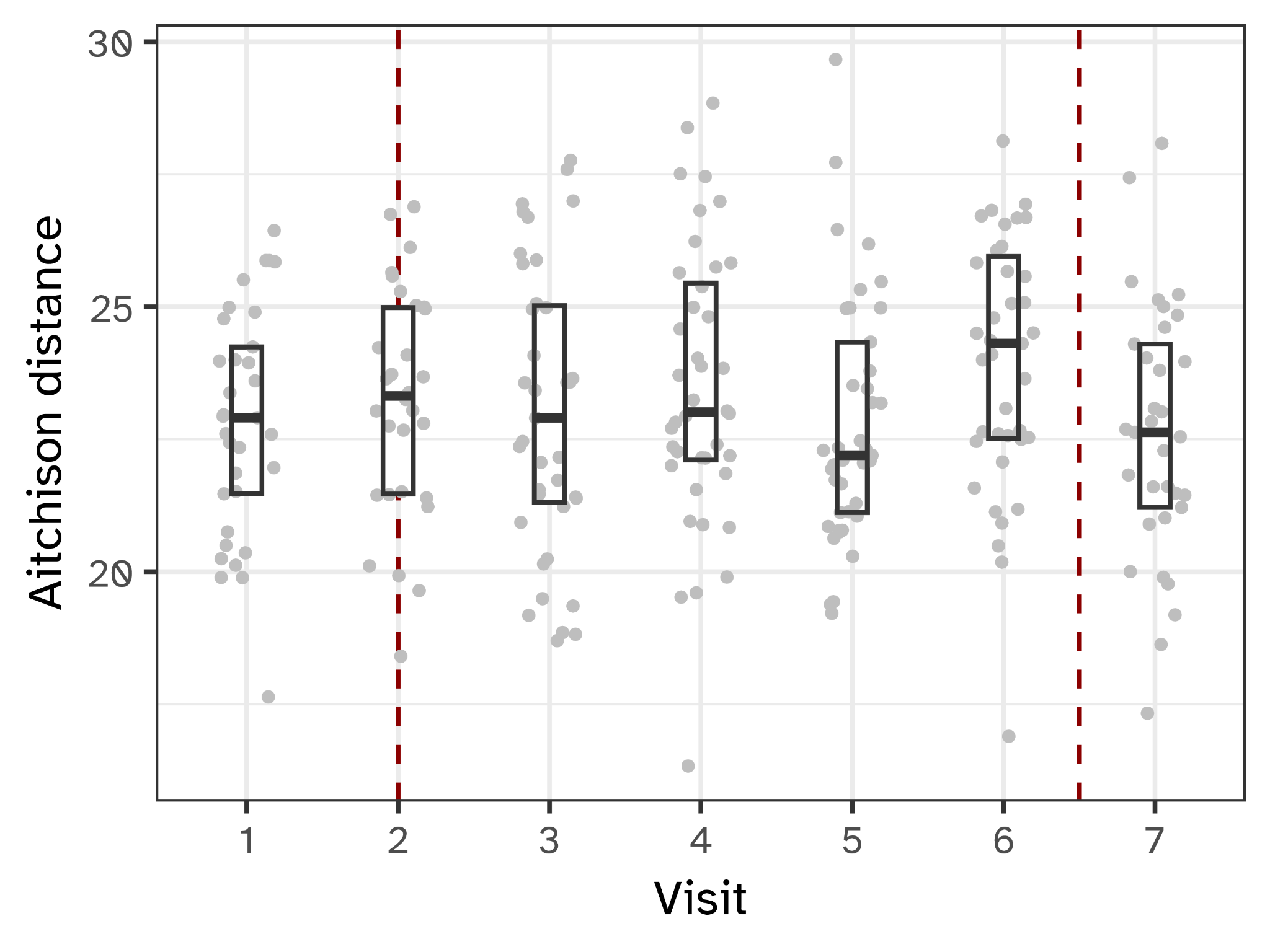


**Supplementary Figure 4.** Aitchinson distance between interproximal plaque (PI), and supragingival plaque (PL), calculated at each timepoint for each individual. The boxplots display the average value, and 25 -75 % percentile. Individual points are shown in gray. Visits 1 and 2 - baseline, visit 3 - day 2, visit 4 - day 5, visit 5 - day 9 and visit 6 - day 14 of experimental gingivitis, visit 7 - day 7 of the resolution phase.


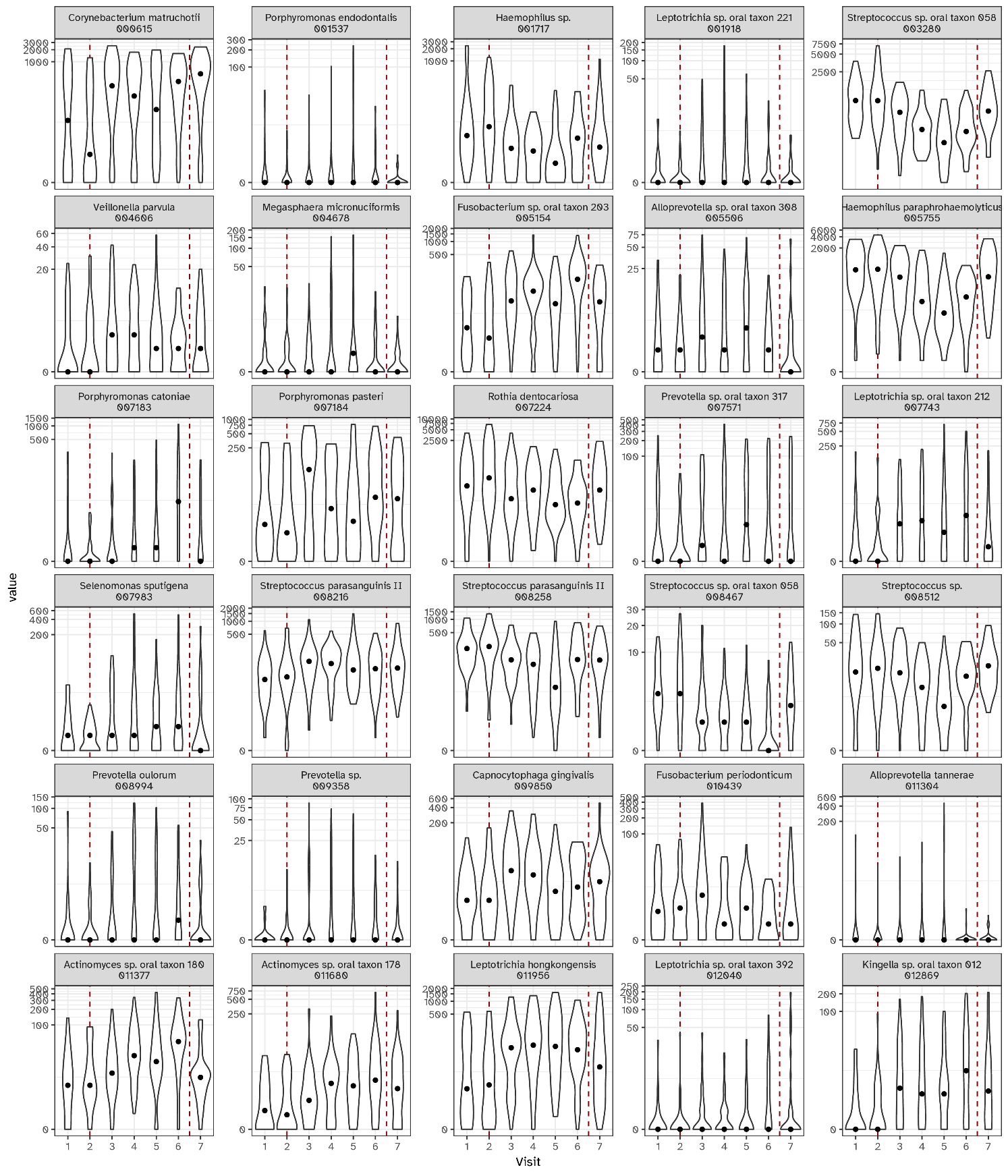


**Supplementary Figure 5.** Violin plot showing the distribution of MED counts (log₂ scale) identified through the Elastic Net model as discriminative for each time point during the gingivitis intervention period in the supragingival plaque (PL) niche. The plot visualizes the variation and density of MED counts across visits, with the log₂ transformation applied to better illustrate differences in magnitude. This figure corresponds to Figure 8, which provides a detailed heatmap of these MEDs, including their taxonomic assignments and relative abundances.. Visits 1 and 2 - baseline, visit 3 - day 2, visit 4 - day 5, visit 5 - day 9 and visit 6 - day 14 of experimental gingivitis, visit 7 - day 7 of the resolution phase.


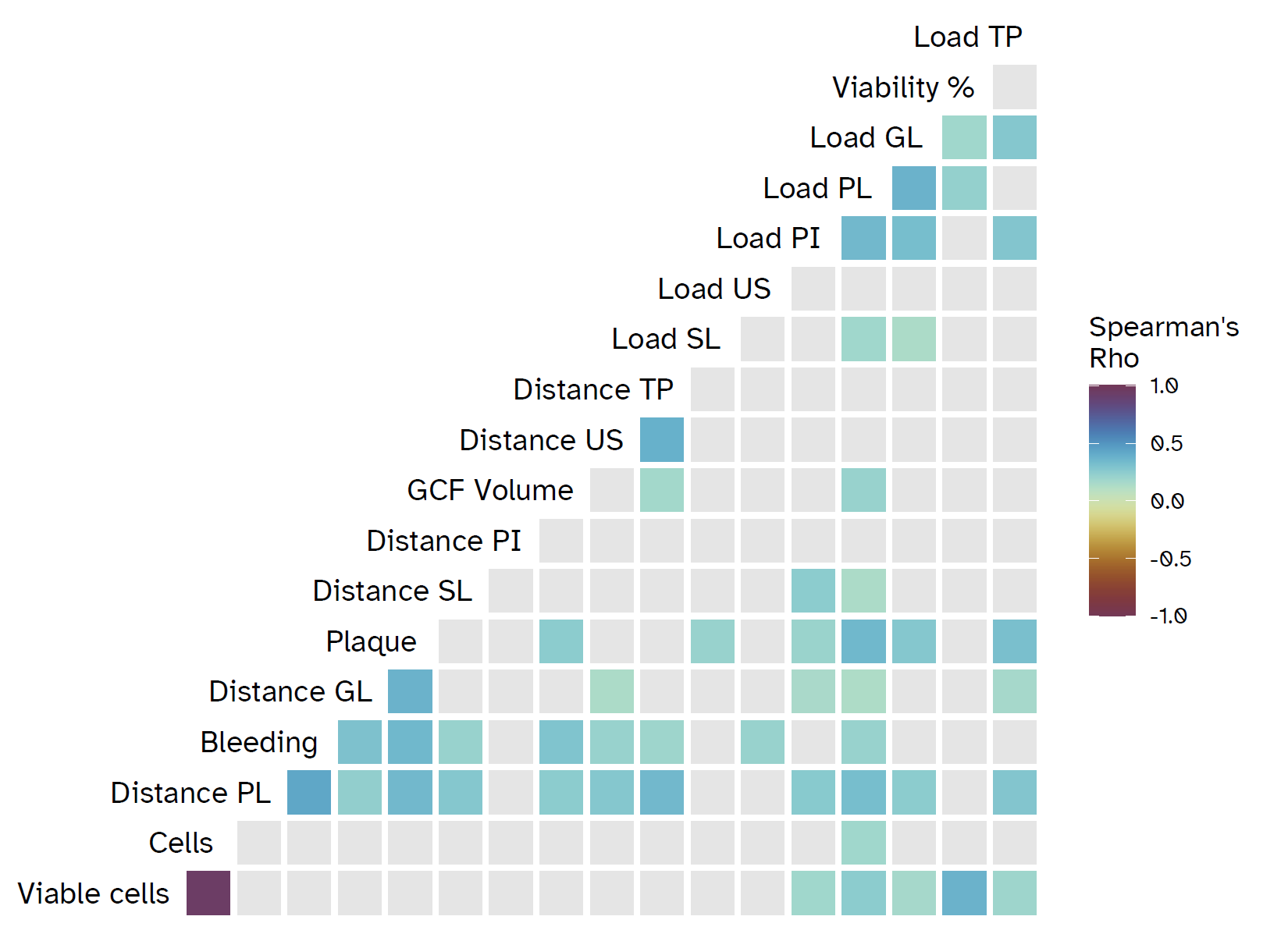
**Supplementary Figure 6**. Correlation matrix heatmap of 18 significant features. Spearman's correlation coefficient was used to compute the relevance and redundancy of the features. Shown are the significant (Spearman Rho P<0,05) correlations of oPMN cell counts (cells), viable oPMN counts (Viable cells), and viable fraction of oPMNs (Viability %), BOMP (bleeding) and plaque percentages, GCF volume along with microbiome compositional change as determined by the Aitchison’s distances (Distance) between baseline (day 0) and completion of the experimental gingivitis period (day 14) and for the bacterial load (Load) in biofilm samples collected for the lower jaw gingiva (distance GL), supragingival plaque (distance PL), subgingival plaque (distance SL), posterior tongue (distance TP) and unstimulated saliva (distance US)
